# Randomness certification in a quantum network with independent sources

**DOI:** 10.1126/sciadv.aea8571

**Published:** 2026-01-23

**Authors:** Giorgio Minati, Giovanni Rodari, Emanuele Polino, Francesco Andreoli, Davide Poderini, Rafael Chaves, Gonzalo Carvacho, Fabio Sciarrino

**Affiliations:** ^1^ Dipartimento di Fisica, Sapienza Università di Roma, P.le Aldo Moro 5, I-00185 Roma, Italy.; ^2^ Centre for Quantum Dynamics and Centre for Quantum Computation and Communication Technology, Griffith University, Yuggera Country, Brisbane, Queensland 4111, Australia.; ^3^ ICFO–Institut de Ciències Fotòniques, The Barcelona Institute of Science and Technology, 08860 Castelldefels, Spain.; ^4^ International Institute of Physics, Federal University of Rio Grande do Norte, 59078-970, Natal, Brazil.; ^5^ Università degli Studi di Pavia, Dipartimento di Fisica, QUIT Group, via Bassi 6, 27100 Pavia, Italy.; ^6^ School of Science and Technology, Federal University of Rio Grande do Norte, 59078-970 Natal, Brazil.

## Abstract

Randomness certification is a foundational and practical aspect of quantum information science, essential for securing quantum communication protocols. Traditionally, these protocols have been implemented and validated with a single entanglement source, as in the paradigmatic Bell scenario. However, advancing these protocols to support more complex configurations involving multiple entanglement sources is key to building robust architectures and realizing large-scale quantum networks. Here, we show how to certify randomness in an entanglement-teleportation experiment, the building block of a quantum repeater displaying two independent sources of entanglement. Using the scalar extension method, we address the challenge posed by the nonconvexity of the correlation set, providing effective bounds on an eavesdropper’s knowledge of the shared secret bits. Our theoretical model characterizes the certifiable randomness within the network and is validated through the analysis of experimental data from a photonic quantum network.

## Teaser

Device-independent randomness is certified within a quantum network and experimentally validated on a photonic platform.

## INTRODUCTION

Quantum nonlocality has captivated scientific interest since the seminal contributions of Einstein *et al.* (*1*) and later Bell (*2*). These foundational studies have prompted extensive investigations into the limitations of local hidden variable theories, which fail to explain the predictions of quantum theory (*3*, *4*). In parallel, advances in quantum information theory have revealed that these nonclassical properties provide essential resources for practical applications such as distributed computing (*5*, *6*) and cryptographic protocols (*7*–*10*).

The nonclassical nature revealed by violations of Bell inequalities serves as the foundation for secure randomness generation and certification, specifically by enabling eavesdropper-secure random bit strings through measurements on a physical system (*11*–*16*). This task can be achieved in a device-independent (DI) framework and has been explored theoretically and experimentally (*17*–*22*), predominantly within the paradigmatic Bell’s scenario, where two distant parties perform local measurements on a shared entangled resource. In this case, the secure randomness that can be generated is quantified using the concept of guessing probability (*13*, *23*), which represents the probability that an external agent, such as an eavesdropper, can correctly predict the measurement results based on the observed output statistics. It has been shown that whenever nonclassicality is manifested through the violation of a Bell inequality, a nonzero amount of randomness can be certified (*11*, *13*, *24*). Similar studies have investigated variations of the bipartite scenario (*12*, *14*–*16*, *24*, *25*) and other configurations, such as Bell-like (*26*, *27*) or broadcasting (*28*) three-party networks and the instrumental scenario (*22*). All of these scenarios share the common feature of involving a single shared source of correlations between distant parties.

Notwithstanding, identifying nonclassicality in scenarios with multiple independent sources is crucial for both foundational research and practical applications in quantum technologies (*29*–*40*). These multisource configurations are essential building blocks for scalable, long-range quantum communication networks. Within the network framework (*29*), independent sources generate a complex, nonconvex set of correlations, making randomness certification particularly challenging. Consequently, existing methods for certifying randomness have shown limited effectiveness when applied to these intricate network structures (*41*–*43*).

Here, we address this challenge by leveraging the scalar extension technique (*44*) to establish a robust framework for randomness certification in quantum networks. To illustrate the general method, we focus on the network underlying the entanglement swapping experiment (*45*), composed of two independent entanglement sources also known as the bilocal scenario (*46*). In particular, this network structure allows for two distinct eavesdropping strategies. For both strategies, we demonstrate that source independence enables the certification of up to 1.41 bits of randomness between the network’s outer nodes—a figure that surpasses the 1.23 bits certified by the maximal violation of the Clauser–Horne–Shimony–Holt (CHSH) inequality (*14*). Furthermore, we apply our framework to certify randomness in the experimental bilocal scenario (*47*), thus demonstrating the feasibility of certifying randomness against eavesdropping threats in operational quantum networks.

## RESULTS

### Randomness in the Bell scenario

**Fig. 1.**
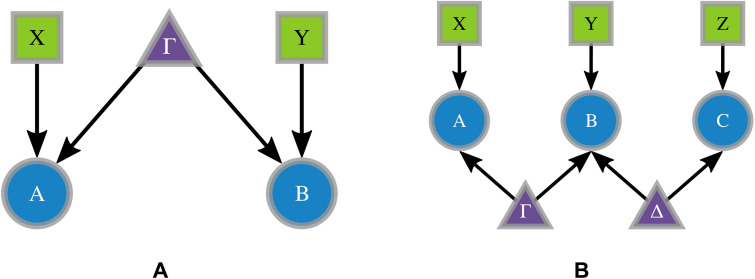
Representation of different causal structures. Directed acyclic graphs (DAGs) represent different causal structures, and the nodes in the graph represent the relevant random variables with arrows accounting for their causal relations. There are three different kinds of nodes: sources of correlations represented either by hidden variables or quantum states (purple triangles), measurement settings (green squares), and measurement outcomes (blue circles). (**A**) Bipartite model with one entangled source. (**B**) Tripartite scenario with two independent sources, accounting for the bilocal hidden variable model.

Bell’s theorem (*3*) is a no-go theorem proving the impossibility of reproducing the predictions of quantum theory within the classical causal model depicted in Fig. 1A. If we consider two parties *A* and *B* performing local measurements on subsystems of a bipartite state $\rho_{\mathrm{AB}}$ produced by a single common source, then the output probabilities predicted by the quantum theory are

$$p_{Q}\left(\mathrm{ab}|\mathrm{xy}\right)=\text{Tr}\left(A_{a}^{x}⊗B_{b}^{y}\cdot \rho_{\mathrm{AB}}\right)$$ (1)

For a suitable choice of an entangled state $\rho_{\mathrm{AB}}$ and operators $\left\{A_{a}^{x},B_{b}^{y}\right\}$, this distribution cannot be described by the classical causal model in Fig. 1A, which implies its incompatibility with a hidden variable model given by

$$p_{L}\left(\mathrm{ab}|\mathrm{xy}\right)=\sum_{\lambda }p\left(a|x,\lambda \right)p\left(b|y,\lambda \right)p\left(\lambda \right)$$ (2)

A notable property of such nonclassical distributions is the possibility to certify that the correlations established between parties *A* and *B* cannot be shared with a third party (*13*). This certification relies on minimal assumptions that an eavesdropper (*E*) has access to an extended quantum state $\rho_{\mathrm{ABE}}$ and that the laboratories in which *A* and *B* carry out their measurements are secure. By further assuming that the eavesdropper’s measurement procedure is described by positive operator-valued measure (POVM) operators $E_{e}$, the information accessible to the eavesdropper should arise from a joint quantum distribution

$$p\left(\mathrm{abe}|\mathrm{xy}\right)=\text{Tr}\left(A_{a}^{x}⊗B_{b}^{y}⊗E_{e}\cdot \rho_{\mathrm{ABE}}\right)$$ (3)

such that *A* and *B* observe a specific probability distribution p(ab∣xy) admitting the realization of Eq. 1.

To bound the amount of information that *E* can extract over the outcomes of *A* and *B*, one considers the guessing probability

$$G\left(\mathrm{AB}|E,\mathrm{xy}\right)=\sum_{\mathrm{ab}}p\left(\mathrm{ab},e=\left(a,b\right)|\mathrm{xy}\right)$$ (4)

The amount of certifiable randomness in a certain scenario is related to the maximum of this quantity achievable with a given realization p(ab∣xy), a problem that can be efficiently solved through semi-definite programming (SDP) techniques as the NPA (Navascués-Pironio-Acín) hierarchy (*48*) under the constraint given by Eq. 3. From the guessing probability, one can readily obtain the amount of certifiable randomness in bits, expressed by the so-called min-entropy (*49*), defined as

$$H_{\text{min}}=-{\text{log}}_{2}\left(G\left(\mathrm{AB}|E,\mathrm{xy}\right)\right)$$ (5)

that can achieve values up to 1.23 bits of randomness in the standard bipartite scenario when bounded by the CHSH inequality (*11*). Within bipartite scenarios, several approaches have been explored to increase the certifiable randomness, reaching up to 2 bits per round. For instance, this value can be obtained at the expense of reduced robustness to noise (*14*), by considering bipartite scenarios with additional inputs (*50*), by using more general positive operator-valued measurements (*15*), or—in the standard case of dichotomic inputs and outputs and for CHSH values in the range $\left[2,3\sqrt{3}/2\right]$—by using so-called “tilted” Bell inequalities (*51*). In this latter approach, as in (*52*), other figures of merit different from $H_{\text{min}}$ have also been considered.

### Randomness certification in the bilocal scenario

Building on the concept of randomness in the standard Bell scenario, several works have addressed its variations (*12*, *14*–*16*, *24*, *25*) and other Bell-like scenarios of relevance (*22*, *26*–*28*). Although scenarios involving multiple independent sources of correlations are crucial for future applications, the challenge of randomness certification in these quantum networks (*29*) remains almost unexplored (*41*–*43*). In this context, the bilocal scenario (*46*), depicted in Fig. 1B, plays a prominent role because it is the underlying causal structure of entanglement swapping (*45*, *53*), an essential protocol for quantum repeaters (*54*, *55*) and long-distance communication networks (*56*, *57*). It consists of two independent sources distributed among three parties: Two of them receive a single subsystem coming, respectively, from $\rho_{{\mathrm{AB}}_{1}}$ and $\rho_{B_{2}C}$, while the central node holds two independent subsystems coming from both sources. Each of the parties carries out local measurements by independently choosing among settings described by the variables {X,Y,Z}, producing outcomes denoted as {A,B,C}, with a probability distribution of measurements outcomes given by

$$p\left(\mathrm{abc}|\mathrm{xyz}\right)=\text{Tr}\left(\rho_{\mathrm{ABC}}\cdot A_{a|x}⊗B_{b|y}⊗C_{c|z}\right)$$ (6)

Here, $\rho_{\mathrm{ABC}}=\rho_{{\mathrm{AB}}_{1}}⊗\rho_{B_{2}C}$ encodes the source independence and $\left\{A_{a|x},B_{b|y},C_{c|z}\right\}$ define the measurements described, in general, by POVM operators. Crucially, the scenario described above cannot be trivially reduced to a configuration consisting of two independent bipartite scenarios (*58*, *59*). Two key features distinguish the bilocal scenario: the underlying causal structure and the range of admissible measurement strategies. Notably, the bilocal framework permits an input-less central node capable of performing entangled measurements. Therefore, compared to separate bipartite configurations, it requires less input randomness and enables access to a broader set of correlations.

**Fig. 2.**
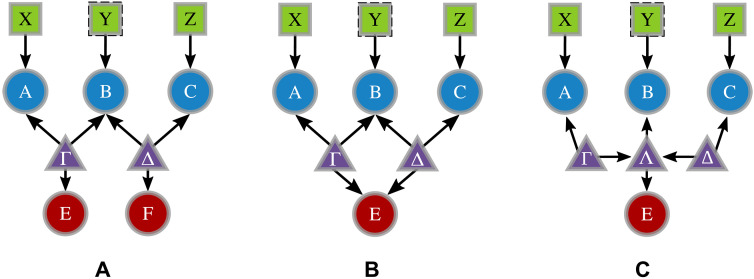
Different eavesdropping strategies within the bilocal scenario. Eavesdropper actions are represented by red circles. (**A**) DE scenario reports a possible eavesdropping strategy within the bilocal scenario, accounting for the case of two distinct agents acting separately on the sources. (**B**) WE scenario reports a single eavesdropper acting on both sources. (**C**) SE scenario is equivalent to additionally supplying Eve with a further latent source. The dashed frame on the setting node *Y* represents the possibility of performing both single- or multiple-setting measurements in the central node.

In contrast to a standard scenario with a single source, quantum networks introduce the constraint of independent sources, which allows multiple ways to model the eavesdropper’s influence. Within our model for randomness certification, we consider three scenarios that may arise in realistic implementations, as illustrated in Fig. 2. First, we examine the possibility of two independent eavesdroppers operating separately at different points in the network, inheriting the limitations of the bilocal scenario, as could occur in networks accessible by multiple users (Fig. 2A). Formally, this means that Eve can perform a POVM $E^{e}⊗F^{f}$ on her share of the state $\rho_{{\mathrm{AB}}_{1}E_{1}}⊗\rho_{{\mathrm{AB}}_{1}E_{2}}$, where $E^{e}$ and $F^{f}$ act only on the parts $E_{1}$ and $E_{2}$, respectively. We will refer to this as the “double-eavesdropper” (DE) scenario. Second, we analyze the case where a single eavesdropper has simultaneous access to both sources, particularly relevant for short-range network connections. This more powerful eavesdropper can measure a general POVM $E^{e}$ on both $E_{1}$ and $E_{2}$, as depicted in Fig. 2B. We will call this the “weak-eavesdropper” (WE) scenario, as a stronger single-eavesdropper configuration is still possible. We can finally consider a scenario within the bilocal network, where the bilocality constraints at nodes *A* and *C* are preserved, but the configuration of the latent variables allows the most general form of eavesdropping attack. We refer to this last scenario as the “strong-eavesdropper” (SE) scenario. We represent this case by introducing an additional latent node Λ affecting both *E* and *B* (see Fig. 2C). Its presence does not affect the independence relation between the other two sources Γ and Δ, nor the one between the outer nodes *A* and *C*, which are the characterizing feature of the bilocal scenario, but rather acts as an extra node that elaborates the incoming signals before sending them to *B* and *E*. In this scenario, the adversary, Eve, is granted access to the same quantum state received by node *B*, allowing her, in principle, to perform measurements compatible with Bob’s settings. This causal structure permits Eve to apply measurement strategies that commute with Bob’s projectors, allowing her to obtain information correlated with Bob’s outcomes without altering the observed probability distribution. However, it should be noted that this does not always guarantee Eve full knowledge of Bob’s measurement results, as the ability to perfectly guess Bob’s outcomes depends on additional constraints such as measurement commutation and state projections that preserve the statistics. While our security analysis adopts the SE model as the conservative, worst-case adversary, we also report the results of the WE and DE assumptions, given their relevant connection to the topology of the bilocal causal structure (independent sources and constrained access), which can be physically enforced in specific deployments. Crucially, the SE bounds always remain valid and provide a lower bound for the WE and DE scenarios, providing a worst-case benchmark for the cases where the weaker-adversary assumptions were to fail.

Note that while the WE and SE scenarios are equivalent when all variables are classical, in the quantum case, there can be a difference. This is related to the known fact that the usual classical exogenization procedures do not work for quantum latent variables with incoming edges (*42*, *60*). Because any eavesdropping strategy, including WE, can also be implemented in the SE case, the certified randomness in the latter scenario will always serve as a lower bound for the former. The increasing generality of the eavesdropping strategies under consideration leads to the inclusion relations

$$Q_{\text{DE}}\subseteq Q_{\text{WE}}\subseteq Q_{\text{SE}}$$

where $Q_{S}$ denotes the set of quantum distributions ${\left\{p\left(\text{abce}|\mathrm{xyz}\right)\right\}}_{S}$ compatible with the corresponding scenario S∈{DE,WE,SE}. Specifically, any WE strategy can be regarded as a particular case of the SE scenario, in which the node Λ acts merely as a relay, forwarding to *B* and *E* the information coming from Γ and Δ without performing additional processing or measurements. Similarly, the DE scenario can be embedded within the WE or SE scenarios by imposing a tensor product structure on the eavesdropper’s measurements, namely

$$E_{e^{\prime }}^{\left(\text{WE}/\text{SE}\right)}=E_{e}^{\left(\text{DE}\right)}⊗F_{f}^{\left(\text{DE}\right)}$$

where $E_{e}^{\left(\text{DE}\right)}$ and $F_{f}^{\left(\text{DE}\right)}$ represent independent measurement operators associated with the two parts of the eavesdropper’s system in the DE scenario. This tensor product structure restricts correlations in the eavesdropper’s measurements, ensuring the inclusion of the DE distributions within the more general WE or SE frameworks. In summary, these inclusion relations reflect the increasing generality of the eavesdropping models: from DE, which assumes independent measurements on separated subsystems, to WE, where Eve directly receives the states from the two independent sources, and last to the fully general SE scenario with arbitrary joint operations. This hierarchy is essential to understand how assumptions about the eavesdropper’s capabilities affect the set of admissible quantum correlations. In this context, it is important to underline that the WE and SE strategies do not account for the possibility that the eavesdropper can control the states emitted by both sources. In such a case, Eve could introduce correlations between the sources, effectively reducing the causal structure to that of a single-source tripartite scenario—a configuration in which randomness certification has already been extensively studied (*26*, *27*) and does not represent an actual network with independent sources, which is the focus of this manuscript. In the following analysis, we will concentrate mostly on the least and most general eavesdropping strategies, namely, the DE and SE scenarios, while some results specifically pertaining to the WE scenario are reported in section “Tilted strategies for the bilocal scenario.”

Analogously to Eq. 4, one can define the global guessing probability in the bilocal scenario as

$$G\left(\mathrm{ABC}|E,\mathrm{xyz}\right)=\sum_{a,b,c}p\left(\mathrm{abc},e=\left(\mathrm{abc}\right)|\mathrm{xyz}\right)$$ (7)

which, again, represents the overall probability for an eavesdropper to correctly guess measurement outcomes.

In the SE scenario, the information available to Eve can be bounded via the following optimization problem

$$\begin{array}{cc} \underset{p}{\text{max}} & G\left(\mathrm{ABC}|E,\mathrm{xyz}\right)\\ \text{s.t.} & p\left(\text{abce}|\mathrm{xyz}\right)=\text{Tr}\left(\rho_{\text{ABCE}}\cdot A_{a|x}⊗B_{b|y}⊗C_{c|z}⊗E_{e}\right),\\ & p\left(\mathrm{abc}|\mathrm{xyz}\right)=\sum_{e}p\left(\text{abce}|\mathrm{xyz}\right)\\ & \rho_{\text{ABCE}}=\rho_{{\mathrm{AB}}_{1}E_{1}}⊗\rho_{B_{2}{\mathrm{CE}}_{2}} \end{array}$$ (8)

Similarly, in the DE scenario, one can instantiate an analogous optimization problem with the crucial difference that the relevant guessing probability is now given by

$$G\left(\mathrm{ABC}|\mathrm{EF},\mathrm{xyz}\right)=\sum_{a,b,c}p\left(\mathrm{abc},e=\left({\mathrm{ab}}_{0}\right),f=\left(b_{1}c\right)|\mathrm{xyz}\right)$$ (9)

where, as will be discussed below, $b_{0}$ and $b_{1}$ correspond to distinct bits associated to Bob’s outcome. In both situations, one could also focus on the guessing probability corresponding only to the outcomes of the outer nodes, that is

$$G\left(\mathrm{AC}|E,\mathrm{xz}\right)=\sum_{a,c}p\left(\mathrm{ac},e=\left(\mathrm{ac}\right)|\mathrm{xz}\right)$$ (10)

Its importance lies in the fact that the bilocal scenario can be seen as the prototype of a long-range quantum communication architecture, exploiting an intermediate node as a quantum repeater, exactly as it happens in event-ready Bell experiments (*45*, *61*, *62*).

#### Single-output versus multiple-output randomness content

In possible applications where only one device’s output (e.g., *a*) is used for randomness extraction, one evaluates the single-output guessing probability, e.g., G(A∣E,x). However, it holds that

G(A∣E,x)≥G(ABC∣E,xyz) (11)

where

$$\left\{\begin{array}{c} G\left(\mathrm{ABC}|E,\mathrm{xyz}\right)=\sum_{a,b,c}p\left(\mathrm{abc},e=\left(\mathrm{abc}\right)|\mathrm{xyz}\right)\\ G\left(A|E,x\right)=\sum_{a}p\left(a,e=\left(a\right)|x\right) \end{array}\right.$$ (12)

One can write

$$\begin{array}{cc} G\left(A|E,x\right) & =\sum_{a}p\left(a,e=\left(a\right)|x\right)=\\ & =\sum_{a}p\left(a,e=\left(a\right)|\mathrm{xyz}\right)=\\ & =\sum_{\mathrm{abc}}p\left(\mathrm{abc},e=\left(a\right)|\mathrm{xyz}\right)\geq \\ & \geq \sum_{\mathrm{abc}}p\left(\mathrm{abc},e=\left(\mathrm{abc}\right)|\mathrm{xyz}\right)=\\ & =G\left(\mathrm{ABC}|E,\mathrm{xyz}\right) \end{array}$$ (13)

Therefore, it holds that the certifiable randomness per trial (smooth min-entropy) for a single output cannot exceed that for the joint variables (*abc*)

$$\begin{array}{cc} H_{\text{min}}\left(A|E,x\right) & =-{\text{log}}_{2}\left(G(A|E,x)\right)\leq \\ & \leq -{\text{log}}_{2}\left(G(\mathrm{ABC}|E,\mathrm{xyz})\right)=\\ & =H_{\text{min}}\left(\mathrm{ABC}|E,\mathrm{xyz}\right) \end{array}$$ (14)

### A numerical approach for randomness certification

To quantify the amount of certifiable randomness in the bilocal scenario, it is necessary to maximize the guessing probability of an eavesdropper. This probability is defined by the expressions in Eqs. 7 to 10, subject to the constraint of observing a set quantum behavior described as in Eq. 6. The result of this optimization provides an estimate of the certifiable randomness in bits, quantified via the min-entropy, $H_{\text{min}}=-{\text{log}}_{2}\left(G\right)$. However, in network scenarios, the independence of sources results in a nonconvex set of correlations (*59*), rendering standard techniques, such as the NPA hierarchy (*63*), inapplicable. To address this challenge, the scalar extension technique (*44*) was developed. This method adapts the NPA hierarchy to account for the independence among the parties, enabling the optimization problem in Eq. 8 to be reformulated as a hierarchy of SDPs. Further details on the scalar extension method and its application to the bilocal scenario can be found in Materials and Methods and the Supplementary Materials.

To illustrate the general method, we start by considering the scenario depicted in Fig. 1A. Each of the sources in the bilocal network is given by noisy quantum states modeled as

$$\rho_{{\mathrm{AB}}_{1}}=\rho_{B_{2}C}=v|\Psi^{-}\rangle \langle \Psi^{-}|+\left(1-v\right)\frac{1}{4}$$ (15)

where *v* is the visibility parameter (*64*). Concerning the measurement operators, two potential measurement strategies performed by the central node are considered: a single projective measurement on the Bell basis or separable measurements given by $B_{0}=\sigma_{z}⊗\sigma_{z}$ and $B_{1}=\sigma_{x}⊗\sigma_{x}$. We will refer to these two choices using the labels “(1,4)” and “(2,2)”, denoting the number of settings and outputs featured by Bob’s measurements, respectively. In what follows, when the presence of multiple settings for Bob’s measurement is unspecified, the corresponding node will be represented by dashed edges, as depicted in Fig. 2. In turn, the outer node measurements have two possibilities, given by

$$A_{0,1}=C_{0,1}=\frac{\sigma_{z}+{\left(-1\right)}^{\left(0,1\right)}\sigma_{x}}{\sqrt{2}}$$ (16)

**Fig. 3.**
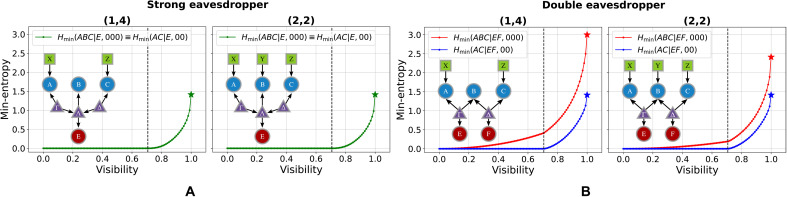
Min-entropy for different configurations of the entanglement-swapping scenario. Taking into account the possible eavesdropping strategies (SE or DE) and measurements performed in the central node [(1,4), (2,2)], we obtain four different configurations. For each of them, we report the min-entropy corresponding to the guessing probability obtained by solving the optimization problem in Eq. 8 using the scalar extension technique. In particular, we plot the min-entropies associated either with the outer (*AC*) or all (*ABC*) parties, as a function of the visibility of the sources state. (**A**) In the strong eavesdropper scenario, both these quantities coincide and are jointly reported as green dots, while in the (**B**) double eavesdropper scenario, they are respectively illustrated as blue and red dots. The stars illustrate the theoretical upper bounds at unitary visibility (see the Supplementary Materials), which are saturated in every configuration of eavesdropping scenarios and measurement choices. The black dashed line shows the threshold visibility below which the states given by the sources, defined in Eq. 15, can no longer violate the CHSH inequality.

Taking these setups into account, we have solved the optimization problem in Eq. 8, over the visibility range v∈[0,1], as reported in Fig. 3 and in Table 1.

**Table 1.** Min-entropy achieved with maximal visibility states. Table accounting for the obtained numerical results. In particular, we report the min-entropies corresponding to states with unitary visibility for all the four configurations of the bilocal scenario that we considered: (1,4) and (2,2) indicate the number of settings and outputs in the central station (Bob).

(*N*_B_, ∣*B*∣_out_)	Strong eavesdropper		Double eavesdropper	
	(1,4)	(2,2)	(1,4)	(2,2)
$H_{\text{min}}\left(\mathrm{ABC}|E\left(F\right),000\right)$	1.41	1.41	3.00	2.41
$H_{\text{min}}\left(\mathrm{AC}|E\left(F\right),00\right)$	1.41	1.41	1.41	1.41

#### Strong eavesdropper (SE) scenario

In the context of the SE scenario for the measurement choices (1,4) and (2,2), we can certify up to ≈1.41 bits of randomness when *v* = 1. This value reaches its theoretical upper bound, as demonstrated by explicitly identifying a potential strategy for Eve. In this specific case of maximal visibility, the strategy involves a nondestructive Bell-state measurement (BSM) of the qubits directed to Bob, followed by a guess of Alice and Charlie’s outcomes based on the expected probability distribution (see the Supplementary Materials). Moreover, Fig. 3A shows that, in the SE scenario, it is possible to certify a nonzero amount of randomness as the visibility of the sources reaches the value $v=1/\sqrt{2}$, known to be the threshold above which a Werner state can violate the CHSH inequality.

#### Double eavesdropper (DE) scenario

Within this scenario, the threshold $v=1/\sqrt{2}$ is no longer valid because a nonzero amount of randomness can still be certified even for $v<1/\sqrt{2}$. In addition, in this scenario, Eve can no longer perform projection measurements on the Bell basis, hence invalidating the previous optimal strategy. This is demonstrated in the numerical results shown in Fig. 3B, where we achieve guessing probabilities as low as $G_{\left(2,2\right)}^{v=1}\left(\mathrm{ABC}|\mathrm{EF},\mathrm{xz}\right)=0.1875$ and $G_{\left(1,4\right)}^{v=1}\left(\mathrm{ABC}|\mathrm{EF},\mathrm{xz}\right)=0.125$, meaning that up to ≈2.41 and ≈3 bits of randomness can be certified for *v* = 1 in the (2,2) and (1,4) measurement settings, respectively. An overview of the min-entropy achieved with maximal visibility states in the considered scenarios is reported in Table 1. Notably, under the assumption of independent eavesdroppers, a nonzero amount of certifiable randomness is observed across the entire range of visibilities in the scenario where the outcomes of all three nodes are guessed. While the randomness generated in this process originates from a combination of classical uncertainty and quantum correlations, this result might be valuable in practical scenarios where the assumption of eavesdropper independence is reasonable. In particular, the eavesdroppers target also the central node’s random variable *b*, which, in both the (2,2) and (1,4) measurement strategies, depends jointly on the latent variables Γ and Λ. However, because in the DE scenario (Fig. 2A) E(F) and Γ(Λ) are independent, neither *E* nor *F* can perfectly infer *b*, giving rise to a classical contribution to the total randomness. It is worth highlighting that both results align with the intuitive observation that the independence of the eavesdroppers prevents them from collaboratively acting on the global system. This restriction inhibits the application of the Bell projection strategy on the central qubits, thereby limiting their predictive capabilities. This classical randomness would not be present in the case where the separate eavesdroppers are allowed to perform classical postprocessing of their data, a different scenario that cannot be treated simply with the scalar extension technique. To summarize, in the visibility range $v\in \left[0,1/\sqrt{2}\right]$, the locality of the source states implies that the certified randomness only comes from the independence constraint; hence, its origin is inherently classical, while for higher visibilities, quantum correlations also play a role. It is worth underlining that this coexistence of both classical and quantum randomness has no counterpart in single-source scenarios, as it is inherently rooted in the network topology arising from the presence of independent sources.

Special attention should be given to the certifiable randomness generated at the outer nodes, as this may represent the key figure of merit in long-distance communication scenarios where the central node functions solely as a repeater. Notably, the numerical results obtained, along with the theoretical upper bounds derived (see the Supplementary Materials), indicate that the amount of randomness reaches 1.41 bits for all combinations of measurement choices [(1,4) or (2,2)] and attack strategies (SE or DE). This exceeds the typical value of 1.23 bits achieved through the violation of the CHSH inequality in a bipartite Bell scenario (*11*), a consequence of the underlying causal structure, that is, it follows from the assumption that the correlations in the network are mediated by two independent sources. Moreover, we underline how such an advantage remains meaningful despite the greater experimental complexity of a realization of a bilocal network. In practical implementations, one might be unable to use a single source to distribute signals, such as in the aforementioned case of long-distance communication scenarios with an intermediate repeater. In such a situation, then the underlying structure is intrinsically constrained to be a bilocal network. As discussed in the Supplementary Materials, Eve’s optimal strategy involves projecting Bob’s state onto the Bell basis, thereby gaining complete knowledge of his measurement outcome. Crucially, the effective state shared between nodes *A* and *C* becomes one of the Bell states, conditioned on the result of Bob’s initial projection. Any further projection on this state by Eve is prohibited, as it would disturb the probability distribution and thus signal the presence of an eavesdropper, a fundamental distinction from the bipartite scenario. This explicit strategy demonstrates that the bilocal scenario allows to certify randomness from its outer nodes even if the resulting distribution observed between these nodes does not enable the violation of any Bell inequality, showing that the network topology plays an active role in the achievable randomness in a causal network.

### Validation on experimental data

**Fig. 4.**
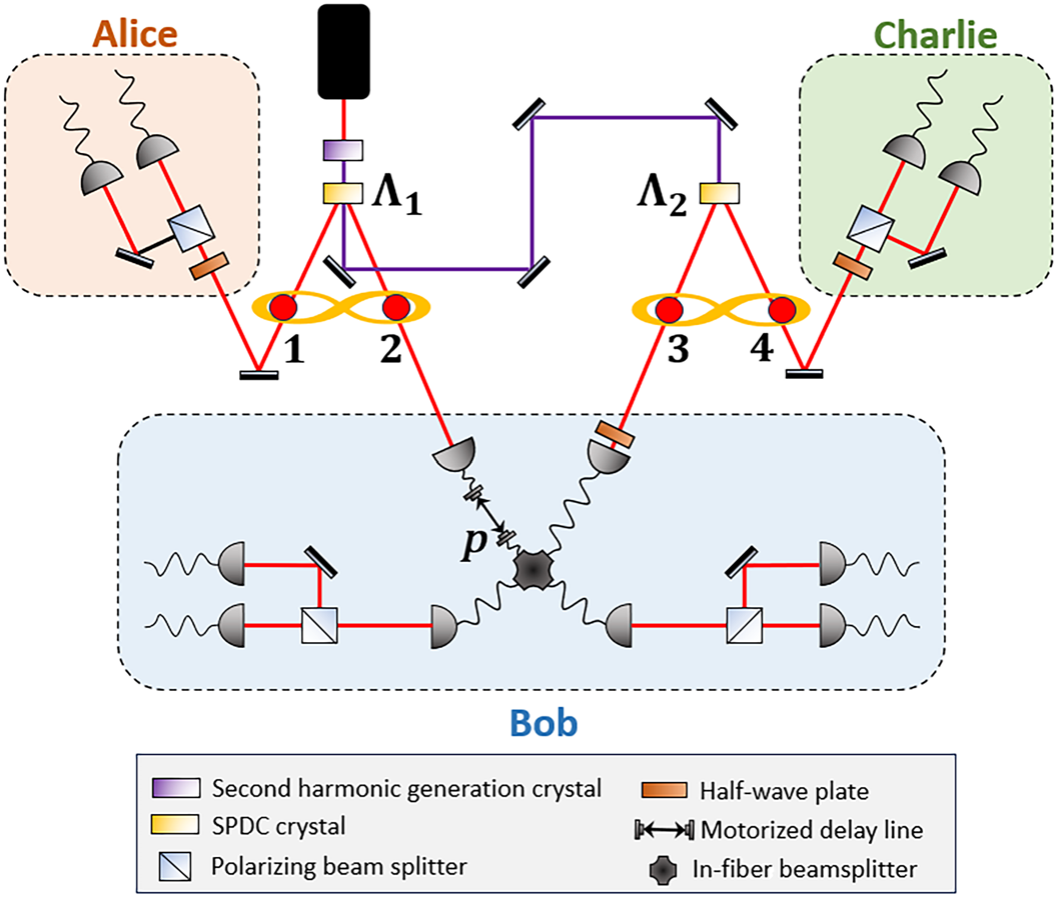
Experimental setup implementing the entanglement swapping network. Two polarization-entangled photon pairs are generated via spontaneous parametric down-conversion (SPDC) in two separated nonlinear crystals. Photons 2 and 3, one from each source, are directed to the central node Bob, while photon 1 (4) is directed to Alice (Charlie). The measurement performed in the central node is fixed and can either discriminate between $|\Psi^{-}\rangle$ and $|\Psi^{+}\rangle$ or between $|\Phi^{-}\rangle$ and $|\Phi^{+}\rangle$, depending on the configuration of the half-wave plate of Bob’s station.

To showcase a practical application of our method, we apply it to analyze the experimental data from (*47*) that uses the photonic setup illustrated in Fig. 4 to provide the first randomness certification of nonlocal correlations within the bilocal scenario. In this setup, two nonlinear crystals generate entangled photon pairs, serving as independent sources of quantum correlations. Alice’s and Charlie’s measurements are performed with polarization analyzers. In addition, a partial BSM is achieved through interference at an in-fiber beamsplitter, where a delay line adjusts the indistinguishability of the incoming photons. Our BSM at the central node is partial: With linear optics and no ancilla/feedforward, only a subset of Bell states is identified via twofold coincidences, and all other events are labeled as “no BSM” (failure). Consequently, the statistics input to our SDP are computed conditioned on the heralding event acc≡(BSM=success). To preclude sampling bias induced by losses or mode-overlap fluctuations, we adopt the standard fair-sampling assumption, namely that acceptance is independent of the would-be outcomes beyond the declared inputs

Pr(acc=1∣x,y,z,λ,E)=η(x,y,z) (17)

independently from *a*, *b*, and *c*, for any internal variables λ and Eve’s side information *E*. Equivalently

p(a,b,c∣x,y,z,acc=1)=p(a,b,c∣x,y,z) (18)

Under this assumption, conditioning on acc=1 yields a representative subset, and our certified smooth min-entropy $H_{\text{min}}^{\varepsilon }\left(\cdot |\text{pub},E,\text{acc}=1\right)$ is a valid lower bound per accepted trial. We note that, in principle, the closure of all relevant loopholes in the bilocal network scenario would allow for a fully DI certification of the underlying quantum correlations. However, achieving complete loophole closure in these multipartite network configurations remains, to our knowledge, an open experimental challenge (*65*).

To compare the theoretical expectations and the experimental finding, we account for several sources of experimental imperfections: (i) the finite indistinguishability of photons 2 and 3, which directly impacts Bob’s measurements; (ii) an improved noise model that includes both white and colored noise in the quantum state; and (iii) statistical fluctuations, which may cause the data to fall slightly outside the set of valid quantum behaviors. Further details on the experimental model are provided in the Supplementary Materials. In addition, we used the NPA hierarchy, augmented with the scalar extension, to evaluate the certifiable randomness from the experimental data.

#### SE scenario

**Fig. 5.**
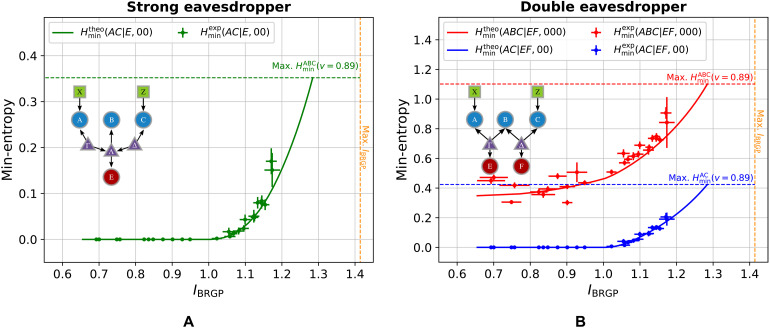
Experimental min-entropy for the strong and double eavesdropper scenarios in the (1,4) measurement setup. The min-entropy, derived from the guessing probability by solving Eq. (8), is shown as a function of the violation of the bilocal inequality $I_{\text{BRGP}}$. Theoretical predictions (solid curves) are compared with experimental data (crosses) for different values of $I_{\text{BRGP}}$, controlled by adjusting the indistinguishability of the photons in the network’s central node. (**A**) In the SE scenario, only the min-entropy of the outer nodes’ outcomes is reported (green crosses and solid curve), as Bob’s outcomes are fully known to the eavesdropper and do not contribute to the certifiable randomness. (**B**) In the DE scenario, $H_{\text{min}}\left(\mathrm{ABC}|\mathrm{EF},000\right)$ (red) and $H_{\text{min}}\left(\mathrm{AC}|\mathrm{EF},00\right)$ (blue) differ and are shown as solid curves and crosses. For both SE and DE cases, the maximum achievable min-entropy within the experimental visibility $v_{\text{exp}}=0.89$ is indicated by dashed lines [green for $H_{\text{min}}^{\left(\text{SE}\right)}\left(\mathrm{AC}|E,000\right)$, red for $H_{\text{min}}^{\left(\text{DE}\right)}\left(\mathrm{ABC}|\mathrm{EF},000\right)$ and blue for $H_{\text{min}}^{\left(\text{DE}\right)}\left(\mathrm{AC}|\mathrm{EF},00\right)$]. The experimental points do not achieve these values because they would require perfectly indistinguishable photons incoming at Bob’s measurement station (further information about the experimental model and the effects of partial indistinguishability are reported in the Supplementary Materials). The orange dashed line represents the maximum violation of $I_{\text{BRGP}}$.

In Fig. 5, we compare the experimental and theoretical min-entropies as a function of the violation of the Branciard-Rosset-Gisin-Pironio bilocal inequality $I_{\text{BRGP}}$, as defined in (*46*), exhibiting excellent agreement. In the SE scenario, the experimental min-entropy on the outer nodes reaches 0.170 ± 0.027 bits compared to its theoretical maximum of 0.35 bits, corresponding to the ideal case where Bob measures completely indistinguishable photons. In this scenario, we do not report the amount of randomness certifiable from all three nodes, as Bob’s outcomes can always be predicted by an eavesdropper in the strong configuration, hence contributing zero bits to the min-entropy.

#### DE scenario

In the context of the DE scenario, the experimental data allow us to certify up to 0.205 ± 0.028 random bits for external nodes *A* and *C* and up to 0.907 ± 0.039 random bits when including all three nodes, while the maximal theoretical predictions achieve 0.424 random bits (external nodes) and 1.10 random bits (all three nodes). This scenario shows very good agreement between the experimental data and the corresponding theoretical model. Minor deviations from the expected behavior can be attributed to experimental fluctuations in the noise parameters across different data acquisitions-fluctuations that are neglected in the theoretical model, which assumes ideal quantum state generation with fixed levels of white and colored noise. These results successfully validate our approach within a practical context and demonstrate that certifying a nonzero amount of secure randomness is feasible in a real-world network implementation.

### Tilted strategies for the bilocal scenario

In the standard Bell scenario, the optimal strategies for randomness certification are not necessarily the ones that are maximally nonlocal (*26*, *51*). We are now going to consider similar strategies for the bilocal scenario using different measurements in the *A* and *C* nodes, inspired by the tilted Bell inequalities, which are known to improve certified randomness in the Bell case (*51*). Specifically, we consider observables of the form

$$A_{0}=\sigma_{z}A_{1}=\text{cos}{\delta \sigma }_{x}-\text{sin}{\delta \sigma }_{z}$$

$$C_{0}=\sigma_{x}C_{1}=\text{cos}{\delta \sigma }_{z}-\text{sin}{\delta \sigma }_{x}$$ (19)

while the central node *B* performs the standard BSM as in the previous case.

#### SE scenario

**Fig. 6.**
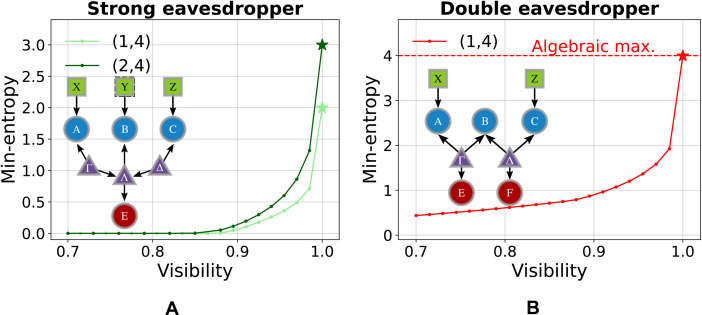
Min-entropy for alternative quantum strategies. Analyze two quantum strategies using tilted Pauli operator: one with a single BSM on *B*, denoted as (1,4) in the figure, and another with two measurement choices on *B*, one of which is a rotated BSM, (2,4) in the figure. (**A**) Min-entropy $H_{\text{min}}\left(\mathrm{ABC}|E\right)$ is shown for both strategies in the SE scenario, where the maximum values reach 3 bits for the (2,4) case and 2 bits for the (1,4) case. (**B**) In the DE scenario, as represented by the corresponding DAG, the (1,4) strategy allows reaching a maximum min-entropy of 4 bits. The stars illustrate the maximum theoretical bound, which is saturated. In particular, the min-entropy attained in the DE scenario reaches its algebraic maximum. This implies that the eavesdroppers do not have any information about the outcomes.

In this case, we find that it is possible to achieve the maximum of 2 bits per round for $H_{\text{min}}\left(\mathrm{AC}|E\right)$ and the same value for $H_{\text{min}}\left(\mathrm{ABC}|E\right)$ (see Fig. 6A). Similarly to the nontilted case, this result can be explained by the fact that Eve can always guess the result of the BSM in the *B* node, as described in the Supplementary Materials. This suggests that the limit of 2 bits could be improved if we introduce a binary measurement setting *Y* for the central node *B*. If we consider a protocol where $B_{0}$ is again the standard BSM while $B_{1}$ projects on the rotated base

$$B_{\theta }=\{\text{cos}\theta |00\rangle +\text{sin}\theta |11\rangle ,\text{cos}\theta |01\rangle +\text{sin}\theta |10\rangle ,$$

sinθ∣00⟩−cosθ∣11⟩,sinθ∣01⟩−cosθ∣10⟩}

we can get up to 3 bits of certified randomness as shown in Fig. 6A.

#### DE and WE scenarios

If, instead, we consider the DE and WE scenarios with the (1,4) strategy, then the restriction on using the same eavesdropping strategy markedly increases the amount of certified randomness. In particular, we can use a self-testing approach (see the Supplementary Materials), in the case of ideal visibility, to certify up to $H_{\text{min}}\left(\mathrm{ABC}|E\left(F\right)\right)=4$ for both the WE and DE scenarios. In such a situation, the eavesdroppers have no information at all about the outcomes, and their best strategy is to uniformly guess them. These findings are corroborated by the numerical results for the DE scenario, as illustrated in Fig. 6B. Last, it is worth mentioning that while the tilted CHSH inequality can certify up to 2 bits of randomness, this is achieved using nearly separable states that exhibit little to no robustness against noise. In contrast, as shown in Fig. 6, the randomness certification inspired by the tilted scenario within the bilocal framework demonstrates greater noise resistance.

## DISCUSSION

The intrinsic randomness of quantum mechanics is fundamental for understanding the nonclassical aspects of the theory. It has several practical applications, including random number generation, randomness certification, and secure quantum communication. Although randomness in Bell-like scenarios—where a single source generates quantum correlations—has been extensively studied and implemented experimentally, extending this framework to quantum networks with multiple independent sources remains largely uncharted. This challenge stems from the complexity of analyzing the nonconvex set of correlations produced by independent sources (*29*, *59*). We have addressed this gap by using the scalar extension method (*44*), which offers a reliable and robust approach to certify randomness within quantum networks.

To illustrate the power and versatility of our approach, we have focused on the entanglement-swapping network, a building block for quantum repeaters and an essential component in scalable quantum networks. This network enables different eavesdropping strategies, depending on whether Eve can access one or both entangled sources. In both scenarios, we demonstrated that up to 1.41 bits of randomness can be certified between the network’s outer nodes, a value that surpasses the 1.23 bits achievable through CHSH inequality violations between these nodes (*12*, *14*). This suggests that the source independence enforced by the network topology can offer an advantage in the randomness certification. When considering all the three network’s nodes, we can exploit tilted measurement strategies to certify up to 4 bits of randomness, meaning that none of the outcomes can be known to potential eavesdroppers in this configuration. In addition, we validated our approach by successfully quantifying the amount of randomness in the experimental data from the first photonic implementation of the bilocal network (*47*).

Overall, we present a thorough analysis of the emergence of randomness in quantum network topologies, with a particular focus on the bilocal network. On one hand, we demonstrate the potential of the scalar extension approach in this context. Because this method can be extended any scenario with causal independence relations between observable nodes of the network, it could be combined with other techniques such as the quantum inflation (*42*) and used for the analysis of other quantum network configurations, such as the star network (*30*, *36*, *66*), the triangle network (*33*, *38*), and the unrelated confounding scenario (*67*). Our results also indicate that refining network configurations or measurement settings can improve randomness generation under realistic adversarial conditions. In addition, they reveal possible structural vulnerabilities, such as node-specific predictability, that may remain hidden in simpler bipartite models. While this work serves as a foundational study, with further improvements, its findings could also find more sophisticated applications in networked quantum systems, including Bernoulli factory processes (*68*–*71*) and blind quantum computation (*72*–*74*), contributing to the advancement of quantum communication architectures where randomness plays a central role.

## MATERIALS AND METHODS

The numerical computation of the amount of randomness within the bilocal scenario is based on the scalar extension technique (*44*), as the standard NPA hierarchy (*63*) cannot capture the causal independence relations that may arise among the network nodes due to the presence of independent sources. In the bilocal scenario, this is evident from the fact that the independence between Alice’s and Charlie’s nodes makes the corresponding probability distribution factorize as $\sum_{b}p\left(a,b,c|x,y,z\right)=p\left(a|x\right)p\left(c|z\right)$. Such an expression is nonlinear and nonconvex, so we can no longer characterize the quantum bilocal set of correlations using standard SDP relaxations.

In the standard NPA hierarchy, a moment matrix of order *k* is constructed as the matrix with entries $\Gamma_{\text{ij}}=\text{Tr}\left(\rho O_{i}O_{j}\right)$, where $O_{i(j)}$ are products of the parties’ measurement operators up to a length *k*. In the limit of k→∞, having Γ≽0 certifies the membership of a given distribution to the set of quantum behaviors.

The main idea of scalar extension is to expand the set of operators that generate the moment matrix by incorporating additional elements derived from the products of actual operators and scalar terms, defined as the expectation values of operators (e.g., terms such as $S_{i}\langle S_{j}\rangle$ or $S_{i}\langle S_{j}\rangle \langle S_{k}\rangle$). These terms must be chosen so that the resulting extended moment matrix $\tilde{\Gamma }$ has factorized entries that encode all the independence relations of the scenario of interest. Hence, a linear expression in the extended moment matrix now suffices to express any independence among the parties, and optimization problems, such as maximizing the guessing probability over the set of bilocal quantum behaviors, can now be cast as SDPs using the scalar extension technique. A crucial feature of the scalar extension technique is its complexity scaling, which does not change with respect to the standard NPA relaxations in single-source multipartite scenarios, regardless of the number of independent sources or connectivity of the causal structure. In both cases, in an N-partite scenario where the parties perform measurements with *m* outcomes and *l* setting, the problem of randomness certification at the *k*-th level of the NPA hierarchy consists in solving an SDP problem with a number of variables scaling as $∼\frac{1}{2}{\left(\mathrm{Nml}\right)}^{2k}$. In conclusion, we believe that this numerical approach constitutes a great resource in the study of quantum networks, as it can be directly extended to any topology that can be characterized by causal independence relations arising among their observable nodes, as the chain and star networks.

## Supplementary Materials

### This PDF file includes:

Supplementary Text Figs. S1 to S5 References

## References

[1] A. Einstein, B. Podolsky, N. Rosen, Can quantum-mechanical description of physical reality be considered complete? Phys. Rev. 47, 777–780 (1935).

[2] J. S. Bell, On the einstein podolsky rosen paradox. Phys. Phys. Fizika 1, 195–200 (1964).

[3] N. Brunner, D. Cavalcanti, S. Pironio, V. Scarani, S. Wehner, Bell nonlocality. Rev. Mod. Phys. 86, 419–478 (2014).

[4] N. Gisin, Physics. Quantum nonlocality: How does nature do it? Science 326, 1357–1358 (2009). 19965747 10.1126/science.1182103

[5] S. Beigi, R. König, Simplified instantaneous non-local quantum computation with applications to position-based cryptography. New J. Phys. 13, 093036 (2011).

[6] H. Buhrman, R. Cleve, S. Massar, R. De Wolf, Nonlocality and communication complexity. Rev. Mod. Phys. 82, 665–698 (2010).

[7] C. Portmann, R. Renner, Security in quantum cryptography. Rev. Mod. Phys. 94, 025008 (2022).

[8] J. Yin, Y.-H. Li, S.-K. Liao, M. Yang, Y. Cao, L. Zhang, J.-G. Ren, W.-Q. Cai, W.-Y. Liu, S.-L. Li, R. Shu, Y.-M. Huang, L. Deng, L. Li, Q. Zhang, N.-L. Liu, Y.-A. Chen, C.-Y. Lu, X.-B. Wang, F. Xu, J.-Y. Wang, C.-Z. Peng, A. K. Ekert, J.-W. Pan, Entanglement-based secure quantum cryptography over 1,120 kilometres. Nature 582, 501–505 (2020). 32541968 10.1038/s41586-020-2401-y

[9] T. Jennewein, C. Simon, G. Weihs, H. Weinfurter, A. Zeilinger, Quantum cryptography with entangled photons. Phys. Rev. Lett. 84, 4729–4732 (2000). 10990782 10.1103/PhysRevLett.84.4729

[10] S. Pirandola, U. L. Andersen, L. Banchi, M. Berta, D. Bunandar, R. Colbeck, D. Englund, T. Gehring, C. Lupo, C. Ottaviani, J. L. Pereira, M. Razavi, J. S. Shaari, M. Tomamichel, V. C. Usenko, G. Vallone, P. Villoresi, P. Wallden, Advances in quantum cryptography. Adv. Opt. Photonics 12, 1012–1236 (2020).

[11] S. Pironio, A. Acín, S. Massar, A. B. de la Giroday, D. N. Matsukevich, P. Maunz, S. Olmschenk, D. Hayes, L. Luo, T. A. Manning, C. Monroe, Random numbers certified by bell’s theorem. Nature 464, 1021–1024 (2010). 20393558 10.1038/nature09008

[12] O. Nieto-Silleras, S. Pironio, J. Silman, Using complete measurement statistics for optimal device-independent randomness evaluation. New J. Phys. 16, 013035 (2014).

[13] A. Acín, L. Masanes, Certified randomness in quantum physics. Nature 540, 213–219 (2016). 27929003 10.1038/nature20119

[14] A. Acín, S. Massar, S. Pironio, Randomness versus nonlocality and entanglement. Phys. Rev. Lett. 108, 100402 (2012). 22463395 10.1103/PhysRevLett.108.100402

[15] A. Acín, S. Pironio, T. Vértesi, P. Wittek, Optimal randomness certification from one entangled bit. Phys. Rev. A 93, 040102 (2016).

[16] E. Woodhead, J. Kaniewski, B. Bourdoncle, A. Salavrakos, J. Bowles, A. Acín, R. Augusiak, Maximal randomness from partially entangled states. Phys. Rev. Res. 2, 042028 (2020).

[17] Y. Liu, Q. Zhao, M.-H. Li, J.-Y. Guan, Y. Zhang, B. Bai, W. Zhang, W.-Z. Liu, C. Wu, X. Yuan, H. Li, W. J. Munro, Z. Wang, L. You, J. Zhang, X. Ma, J. Fan, Q. Zhang, J.-W. Pan, Device-independent quantum random-number generation. Nature 562, 548–551 (2018). 30287887 10.1038/s41586-018-0559-3

[18] S. Gómez, A. Mattar, E. S. Gómez, D. Cavalcanti, O. J. Farías, A. Acín, G. Lima, Experimental nonlocality-based randomness generation with nonprojective measurements. Phys. Rev. A 97, 040102 (2018).

[19] M.-H. Li, X. Zhang, W.-Z. Liu, S.-R. Zhao, B. Bai, Y. Liu, Q. Zhao, Y. Peng, J. Zhang, Y. Zhang, W. J. Munro, X. Ma, Q. Zhang, J. Fan, J.-W. Pan, Experimental realization of device-independent quantum randomness expansion. Phys. Rev. Lett. 126, 050503 (2021). 33605771 10.1103/PhysRevLett.126.050503

[20] L. K. Shalm, Y. Zhang, J. C. Bienfang, C. Schlager, M. J. Stevens, M. D. Mazurek, C. Abellán, W. Amaya, M. W. Mitchell, M. A. Alhejji, H. Fu, J. Ornstein, R. P. Mirin, S. W. Nam, E. Knill, Device-independent randomness expansion with entangled photons. Nat. Phys. 17, 452–456 (2021).

[21] A. J.-M. Seguinard, A. Piveteau, P. Mironowicz, M. Bourennane, Experimental certification of more than one bit of quantum randomness in the two inputs and two outputs scenario. New J. Phys. 25, 113022 (2023).

[22] I. Agresti, D. Poderini, L. Guerini, M. Mancusi, G. Carvacho, L. Aolita, D. Cavalcanti, R. Chaves, F. Sciarrino, Experimental device-independent certified randomness generation with an instrumental causal structure. Commun. Phys. 3, 110 (2020).

[23] R. Colbeck, Quantum and relativistic protocols for secure multi-party computation. arXiv:0911.3814 [quant-ph] (2009).

[24] F. J. Curchod, M. Johansson, R. Augusiak, M. J. Hoban, P. Wittek, A. Acín, Unbounded randomness certification using sequences of measurements. Phys. Rev. A 95, 020102 (2017).

[25] J.-D. Bancal, L. Sheridan, V. Scarani, More randomness from the same data. New J. Phys. 16, 033011 (2014).

[26] E. Woodhead, B. Bourdoncle, A. Acín, Randomness versus nonlocality in the Mermin-Bell experiment with three parties. Quantum 2, 82 (2018).

[27] F. Grasselli, G. Murta, H. Kampermann, D. Bruß, Boosting device-independent cryptography with tripartite nonlocality. Quantum 7, 980 (2023).

[28] E. Polino, L. Villegas-Aguilar, D. Poderini, N. Walk, F. Ghafari, M. T. Quintino, A. Lyasota, S. Rogge, R. Chaves, G. J. Pryde, E. G. Cavalcanti, N. Tischler, S. Slussarenko, Experimental quantum randomness enhanced by a quantum network. arXiv:2412.16973 [quant-ph] (2024).

[29] A. Tavakoli, A. Pozas-Kerstjens, M.-X. Luo, M.-O. Renou, Bell nonlocality in networks. Rep. Prog. Phys. 85, 056001 (2022). 10.1088/1361-6633/ac41bb 34883470

[30] D. Poderini, I. Agresti, G. Marchese, E. Polino, T. Giordani, A. Suprano, M. Valeri, G. Milani, N. Spagnolo, G. Carvacho, R. Chaves, F. Sciarrino, Experimental violation of n-locality in a star quantum network. Nat. Commun. 11, 2467 (2020). 32424194 10.1038/s41467-020-16189-6 PMC7235259

[31] R. Chaves, G. Moreno, E. Polino, D. Poderini, I. Agresti, A. Suprano, M. R. Barros, G. Carvacho, E. Wolfe, A. Canabarro, R. W. Spekkens, F. Sciarrino, Causal networks and freedom of choice in bell’s theorem. PRX Quantum 2, 040323 (2021).

[32] A. Suprano, D. Poderini, E. Polino, I. Agresti, G. Carvacho, A. Canabarro, E. Wolfe, R. Chaves, F. Sciarrino, Experimental genuine tripartite nonlocality in a quantum triangle network. PRX Quantum 3, 030342 (2022).

[33] E. Polino, D. Poderini, G. Rodari, I. Agresti, A. Suprano, G. Carvacho, E. Wolfe, A. Canabarro, G. Moreno, G. Milani, R. W. Spekkens, R. Chaves, F. Sciarrino, Experimental nonclassicality in a causal network without assuming freedom of choice. Nat. Commun. 14, 909 (2023). 36808157 10.1038/s41467-023-36428-w PMC9938195

[34] F. Andreoli, G. Carvacho, L. Santodonato, M. Bentivegna, R. Chaves, F. Sciarrino, Experimental bilocality violation without shared reference frames. Phys. Rev. A 95, 062315 (2017).

[35] N. D’Alessandro, B. Polacchi, G. Moreno, E. Polino, R. Chaves, I. Agresti, F. Sciarrino, Machine-learning-based device-independent certification of quantum networks. Phys. Rev. Res. 5, 023016 (2023).

[36] N.-N. Wang, A. Pozas-Kerstjens, C. Zhang, B.-H. Liu, Y.-F. Huang, C.-F. Li, G.-C. Guo, N. Gisin, A. Tavakoli, Certification of non-classicality in all links of a photonic star network without assuming quantum mechanics. Nat. Commun. 14, 2153 (2023). 37059704 10.1038/s41467-023-37842-w PMC10104853

[37] X.-M. Gu, L. Huang, A. Pozas-Kerstjens, Y.-F. Jiang, D. Wu, B. Bai, Q.-C. Sun, M.-C. Chen, J. Zhang, S. Yu, Q. Zhang, C.-Y. Lu, J.-W. Pan, Experimental full network nonlocality with independent sources and strict locality constraints. Phys. Rev. Lett. 130, 190201 (2023). 37243635 10.1103/PhysRevLett.130.190201

[38] N.-N. Wang, C. Zhang, H. Cao, K. Xu, B.-H. Liu, Y.-F. Huang, C.-F. Li, G.-C. Guo, N. Gisin, T. Kriváchy, M.-O. Renou, Experimental genuine quantum nonlocality in the triangle network. arXiv:2401.15428 [quant-ph] (2024). 10.1103/5hhc-rw3t 42172390

[39] D. J. Saunders, A. J. Bennet, C. Branciard, G. J. Pryde, Experimental demonstration of nonbilocal quantum correlations. Sci. Adv. 3, e1602743 (2017). 28508045 10.1126/sciadv.1602743 PMC5409499

[40] G. Carvacho, E. Roccia, M. Valeri, F. B. Basset, D. Poderini, C. Pardo, E. Polino, L. Carosini, M. B. Rota, J. Neuwirth, S. F. Covre da Silva, A. Rastelli, N. Spagnolo, R. Chaves, R. Trotta, F. Sciarrino, Quantum violation of local causality in an urban network using hybrid photonic technologies. Optica 9, 572–578 (2022).

[41] C. M. Lee, M. J. Hoban, Towards device-independent information processing on general quantum networks. Phys. Rev. Lett. 120, 020504 (2018). 29376705 10.1103/PhysRevLett.120.020504

[42] E. Wolfe, A. Pozas-Kerstjens, M. Grinberg, D. Rosset, A. Acín, M. Navascués, Quantum inflation: A general approach to quantum causal compatibility. Phys. Rev. X 11, 021043 (2021).

[43] P. Sekatski, S. Boreiri, N. Brunner, Partial self-testing and randomness certification in the triangle network. Phys. Rev. Lett. 131, 100201 (2023). 37739349 10.1103/PhysRevLett.131.100201

[44] A. Pozas-Kerstjens, R. Rabelo, Ł. Rudnicki, R. Chaves, D. Cavalcanti, M. Navascués, A. Acín, Bounding the sets of classical and quantum correlations in networks. Phys. Rev. Lett. 123, 140503 (2019). 31702186 10.1103/PhysRevLett.123.140503

[45] M. Z. Żukowski, A. Zeilinger, M. A. Horne, A. K. Ekert, “Event-ready-detectors” Bell experiment via entanglement swapping. Phys. Rev. Lett. 71, 4287–4290 (1993). 10055208 10.1103/PhysRevLett.71.4287

[46] C. Branciard, D. Rosset, N. Gisin, S. Pironio, Bilocal versus nonbilocal correlations in entanglement-swapping experiments. Phys. Rev. A 85, 032119 (2012).

[47] G. Carvacho, F. Andreoli, L. Santodonato, M. Bentivegna, R. Chaves, F. Sciarrino, Experimental violation of local causality in a quantum network. Nat. Commun. 8, 14775 (2017). 28300068 10.1038/ncomms14775 PMC5356073

[48] M. Navascués, S. Pironio, A. Acín, A convergent hierarchy of semidefinite programs characterizing the set of quantum correlations. New J. Phys. 10, 073013 (2008).

[49] S. Pironio, S. Massar, Security of practical private randomness generation. Phys. Rev. A 87, 012336 (2013).

[50] C. Dhara, G. Prettico, A. Acín, Maximal quantum randomness in bell tests. Phys. Rev. A 88, 052116 (2013).

[51] L. Wooltorton, P. Brown, R. Colbeck, Tight analytic bound on the trade-off between device-independent randomness and nonlocality. Phys. Rev. Lett. 129, 150403 (2022). 36269949 10.1103/PhysRevLett.129.150403

[52] R. Bhavsar, S. Ragy, R. Colbeck, Improved device-independent randomness expansion rates using two sided randomness. New J. Phys. 25, 093035 (2023).

[53] M. Zukowski, A. Zeilinger, H. Weinfurter, Entangling photons radiated by independent pulsed sources. Ann. N. Y. Acad. Sci. 755, 91–102 (1995).

[54] K. Azuma, S. E. Economou, D. Elkouss, P. Hilaire, L. Jiang, H.-K. Lo, I. Tzitrin, Quantum repeaters: From quantum networks to the quantum internet. Rev. Mod. Phys. 95, 045006 (2023).

[55] Z.-D. Li, R. Zhang, X.-F. Yin, L.-Z. Liu, Y. Hu, Y.-Q. Fang, Y.-Y. Fei, X. Jiang, J. Zhang, L. Li, N.-L. Liu, F. Xu, Y.-A. Chen, J.-W. Pan, Experimental quantum repeater without quantum memory. Nat. Photonics 13, 644–648 (2019).

[56] Y.-A. Chen, Q. Zhang, T.-Y. Chen, W.-Q. Cai, S.-K. Liao, J. Zhang, K. Chen, J. Yin, J.-G. Ren, Z. Chen, S.-L. Han, Q. Yu, K. Liang, F. Zhou, X. Yuan, M.-S. Zhao, T.-Y. Wang, X. Jiang, L. Zhang, W.-Y. Liu, Y. Li, Q. Shen, Y. Cao, C.-Y. Lu, R. Shu, J.-Y. Wang, L. Li, N.-L. Liu, F. Xu, X.-B. Wang, C.-Z. Peng, J.-W. Pan, An integrated space-to-ground quantum communication network over 4,600 kilometres. Nature 589, 214–219 (2021). 33408416 10.1038/s41586-020-03093-8

[57] S.-K. Liao, W.-Q. Cai, J. Handsteiner, B. Liu, J. Yin, L. Zhang, D. Rauch, M. Fink, J.-G. Ren, W.-Y. Liu, Y. Li, Q. Shen, Y. Cao, F.-Z. Li, J.-F. Wang, Y.-M. Huang, L. Deng, T. Xi, L. Ma, T. Hu, L. Li, N.-L. Liu, F. Koidl, P. Wang, Y.-A. Chen, X.-B. Wang, M. Steindorfer, G. Kirchner, C.-Y. Lu, R. Shu, R. Ursin, T. Scheidl, C.-Z. Peng, J.-Y. Wang, A. Zeilinger, J.-W. Pan, Satellite-relayed intercontinental quantum network. Phys. Rev. Lett. 120, 030501 (2018). 29400544 10.1103/PhysRevLett.120.030501

[58] I. Šupić, J.-D. Bancal, Y. Cai, N. Brunner, Genuine network quantum nonlocality and self-testing. Phys. Rev. A 105, 022206 (2022).

[59] R. Chaves, Polynomial bell inequalities. Phys. Rev. Lett. 116, 010402 (2016). 26799003 10.1103/PhysRevLett.116.010402

[60] D. Centeno, E. Wolfe, Distinguishing quantum causal scenarios with indistinguishable classical analogs: The significance of intermediate latents. Phys. Rev. A 112, 042206 (2025).

[61] B. Hensen, H. Bernien, A. E. Dréau, A. Reiserer, N. Kalb, M. S. Blok, J. Ruitenberg, R. F. L. Vermeulen, R. N. Schouten, C. Abellán, W. Amaya, V. Pruneri, M. W. Mitchell, M. Markham, D. J. Twitchen, D. Elkouss, S. Wehner, T. H. Taminiau, R. Hanson, Loophole-free bell inequality violation using electron spins separated by 1.3 kilometres. Nature 526, 682–686 (2015). 26503041 10.1038/nature15759

[62] W. Rosenfeld, D. Burchardt, R. Garthoff, K. Redeker, N. Ortegel, M. Rau, H. Weinfurter, Event-ready bell test using entangled atoms simultaneously closing detection and locality loopholes. Phys. Rev. Lett. 119, 010402 (2017). 28731745 10.1103/PhysRevLett.119.010402

[63] M. Navascués, S. Pironio, A. Acín, Bounding the set of quantum correlations. Phys. Rev. Lett. 98, 010401 (2007). 17358458 10.1103/PhysRevLett.98.010401

[64] R. F. Werner, Quantum states with einstein-podolsky-rosen correlations admitting a hidden-variable model. Phys. Rev. A 40, 4277–4281 (1989). 10.1103/physreva.40.4277 9902666

[65] Q.-C. Sun, Y.-F. Jiang, B. Bai, W. Zhang, H. Li, X. Jiang, J. Zhang, L. You, X. Chen, Z. Wang, Q. Zhang, J. Fan, J.-W. Pan, Experimental demonstration of non-bilocality with truly independent sources and strict locality constraints. Nat. Photonics 13, 687–691 (2019).

[66] F. Andreoli, G. Carvacho, L. Santodonato, R. Chaves, F. Sciarrino, Maximal qubit violation of n-locality inequalities in a star-shaped quantum network. New J. Phys. 19, 113020 (2017).

[67] P. Lauand, D. Poderini, R. Rabelo, R. Chaves, Quantum non-classicality in the simplest causal network. arXiv:2404.12790 [quant-ph] (2024).

[68] J. Jiang, J. Zhang, X. Sun, Quantum-to-quantum bernoulli factory problem. Phys. Rev. A 97, 032303 (2018).

[69] Y. Liu, J. Jiang, P. Zhu, D. Wang, J. Ding, X. Qiang, A. Huang, P. Xu, J. Zhang, G. Tian, X. Fu, M. Deng, C. Wu, X. Sun, X. Yang, J. Wu, General quantum bernoulli factory: Framework analysis and experiments. Quantum Sci. Technol. 6, 045025 (2021).

[70] F. Hoch, T. Giordani, L. Castello, G. Carvacho, N. Spagnolo, F. Ceccarelli, C. Pentangelo, S. Piacentini, A. Crespi, R. Osellame, E. F. Galvão, F. Sciarrino, Modular quantum-to-quantum bernoulli factory in an integrated photonic processor. Nat. Photonics 19, 12–19 (2025).

[71] G. Rodari, F. Hoch, A. Suprano, T. Giordani, E. Negro, G. Carvacho, N. Spagnolo, E. F. Galvão, F. Sciarrino, Polarization-encoded photonic quantum-to-quantum bernoulli factory based on a quantum dot source. Sci. Adv. 10, eado6244 (2024). 39058770 10.1126/sciadv.ado6244 PMC11777904

[72] A. Broadbent, J. Fitzsimons, E. Kashefi, “Universal blind quantum computation,” in *2009 50th annual IEEE symposium on foundations of computer science* (IEEE, 2009), pp. 517–526. 10.1109/FOCS.2009.36.

[73] B. Polacchi, D. Leichtle, L. Limongi, G. Carvacho, G. Milani, N. Spagnolo, M. Kaplan, F. Sciarrino, E. Kashefi, Multi-client distributed blind quantum computation with the qline architecture. Nat. Commun. 14, 7743 (2023). 38007542 10.1038/s41467-023-43617-0 PMC10676426

[74] B. Polacchi, D. Leichtle, G. Carvacho, G. Milani, N. Spagnolo, M. Kaplan, E. Kashefi, F. Sciarrino, Experimental verifiable multiclient blind quantum computing on a qline architecture. Phys. Rev. Lett. 134, 200603 (2025). 40479690 10.1103/PhysRevLett.134.200603

[75] K. Mattle, H. Weinfurter, P. G. Kwiat, A. Zeilinger, Dense coding in experimental quantum communication. Phys. Rev. Lett. 76, 4656–4659 (1996). 10061348 10.1103/PhysRevLett.76.4656

[76] A. Cabello, A. Feito, A. Lamas-Linares, Bell’s inequalities with realistic noise for polarization-entangled photons. Phys. Rev. A 72, 052112 (2005).

[77] M. O. Renou, J. Kaniewski, N. Brunner, Self-testing entangled measurements in quantum networks. Phys. Rev. Lett. 121, 250507 (2018). 30608820 10.1103/PhysRevLett.121.250507

[78] Y. Li, Y. Xiang, X.-D. Yu, H. C. Nguyen, O. Gühne, Q. He, Randomness certification from multipartite quantum steering for arbitrary dimensional systems. Phys. Rev. Lett. 132, 080201 (2024). 38457732 10.1103/PhysRevLett.132.080201

