# Supplementary material for: Randomness certification in a quantum network with independent sources
Source: Sci Adv. 2026 Jan 23;12(4):eaea8571. doi: 10.1126/sciadv.aea8571 (PMC12829563; doi:10.1126/sciadv.aea8571)
Supplement: Supplementary file 1 — Supplementary Text Figs. S1 to S5 References [file sciadv.aea8571_sm.pdf]

Science Advances
AAAS

Supplementary Materials for

Randomness certification in a quantum network with independent sources

Giorgio Minati *et al.*

Corresponding author: Gonzalo Carvacho, gonzalo.carvacho@uniroma1.it

Sci. Adv. **12**, eaea8571 (2026)
DOI: 10.1126/sciadv.aea8571

This PDF file includes:

Supplementary Text
Figs. S1 to S5
References

I. NOISE MODELING OF THE EXPERIMENTAL APPARATUS

When modeling the experimental distributions, we have to take into account two key aspects: the imperfect indistinguishability of the two incoming photons at Bob's measurement station and the presence of noise in the states generated by the sources. In the following, we will address each of these effects.

A. Partial indistinguishability

The partial indistinguishability directly affects the Bell State Measurement (BSM), since it mixes the detection of the Bell states $|\Psi^-\rangle \leftrightarrow |\Psi^+\rangle$ and $|\Phi^-\rangle \leftrightarrow |\Phi^+\rangle$, but it does not mix states belonging to different categories (*75*). We can model such an effect by substituting the projectors onto the Bell states with suitable effective POVMs:

$$\begin{aligned}
|\Psi^-\rangle\langle\Psi^-| &\longrightarrow \hat{F}_1 = \frac{1+p}{2}|\Psi^-\rangle\langle\Psi^-| + \frac{1-p}{2}|\Psi^+\rangle\langle\Psi^+|,\\
|\Psi^+\rangle\langle\Psi^+| &\longrightarrow \hat{F}_2 = \frac{1-p}{2}|\Psi^-\rangle\langle\Psi^-| + \frac{1+p}{2}|\Psi^+\rangle\langle\Psi^+|,\\
|\Phi^-\rangle\langle\Phi^-| &\longrightarrow \hat{F}_3 = \frac{1+p}{2}|\Phi^-\rangle\langle\Phi^-| + \frac{1-p}{2}|\Phi^+\rangle\langle\Phi^+|,\\
|\Phi^+\rangle\langle\Phi^+| &\longrightarrow \hat{F}_4 = \frac{1-p}{2}|\Phi^-\rangle\langle\Phi^-| + \frac{1+p}{2}|\Phi^+\rangle\langle\Phi^+|,
\end{aligned} \tag{S1}$$

where the parameter $p \in [0,1]$ quantifies the indistinguishability of the two photons. In particular, when $p = 0$ the photons are distinguishable and the success rate of the measurements is 50%, while in the case of indistinguishable photons ($p = 1$) the effective POVMs $\hat{F}_{1,2,3,4}$ coincide with the BSM. Experimentally, this parameter can be continuously tuned using a motorized delay line.

B. Noise in the SPDC sources of quantum states

The quantum states generated through Spontaneous Parametric Down-Conversion (SPDC) sources, in our case, singlet states $|\Psi^-\rangle$, are affected by two distinct types of noise (*76*):

- *White noise:* corresponds to an isotropic depolarization of the state, thus mixing the singlet state with a completely mixed state:

$$\rho = v\,|\Psi^-\rangle\langle\Psi^-| + (1-v)\frac{\mathbb{1}}{4}, \tag{S2}$$

  where $\mathbb{1}$ is the identity matrix and v represents the visibility of the state.

- *Colored noise:* corresponds to a depolarization over a preferred direction, thus resulting in a statistical superposition of the singlet and a mix of $|\Psi^-\rangle$ and $|\Psi^+\rangle$:

$$\rho = v\,|\Psi^-\rangle\langle\Psi^-| + \frac{1-v}{2}\left(|\Psi^-\rangle\langle\Psi^-| + |\Psi^+\rangle\langle\Psi^+|\right). \tag{S3}$$

Therefore, we can model a state featuring both white and colored noise as:

$$\rho_{v,c} = v\,|\Psi^-\rangle\langle\Psi^-| + (1-v)\left[\frac{1}{2}c\left(|\Psi^-\rangle\langle\Psi^-| + |\Psi^+\rangle\langle\Psi^+|\right) + (1-c)\frac{\mathbb{1}}{4}\right], \tag{S4}$$

where v is the overall visibility of the state, while c represents the fraction of colored noise over the total noise.

C. Randomness estimation in presence of experimental noise

To understand how the amount of certified randomness can be affected by the experimental imperfections, we solved the guessing probability maximization problem for different regimes of noise. In particular, we considered the bilocality scenario in the case in which two eavesdroppers separately act on the sources and Bob is carrying out Bell state

measurements. Hence, the numerical evaluation of the guessing probability in the presence of experimental noise consists of solving the following optimization problem:

$$\begin{aligned} \max \quad & G(A(B)C|EF, x(y)z) \quad \text{s.t.} \\ p(abc|xyz) &= \mathrm{Tr}\Big(\rho^{\exp}_{ABC} \cdot A_{a|x} \otimes B^{\exp}_{b|y} \otimes C_{c|z}\Big), \\ p(abc|xyz) &= \sum_{ef} p(abc, ef|xyz), \end{aligned} \tag{S5}$$

where $\rho^{\exp}_{ABC} = \rho^{v,c}_{AB} \otimes \rho^{v,c}_{BC}$ with $\rho^{v,c}$ given by the noisy model of the experimentally generated quantum states reported in Eq.S4, while $B^{\exp}_{b|y}$ corresponds to the effective Bell state measurements described in Eq.S1.

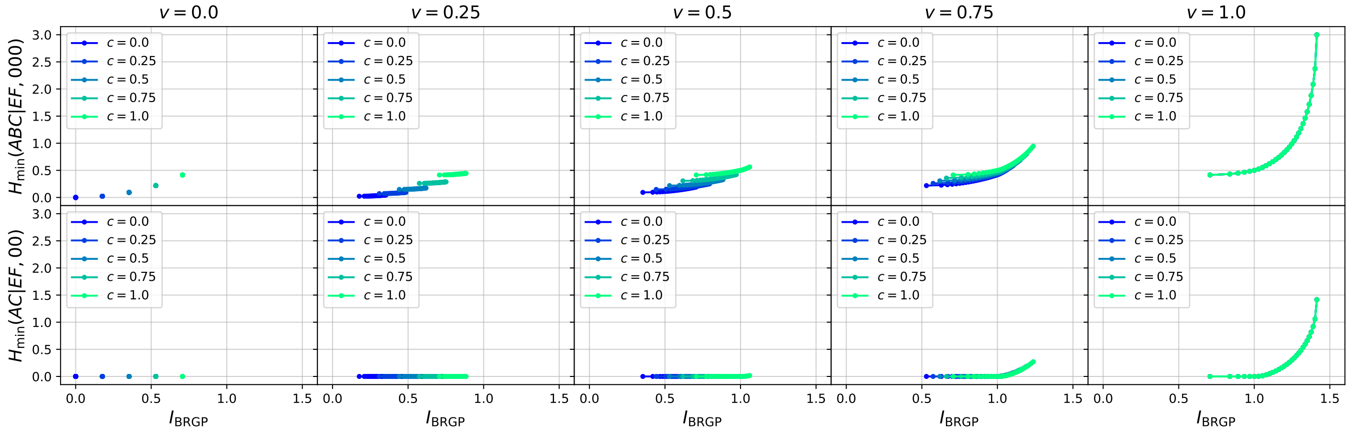

Supplementary Figure 1. **Min-entropy in presence of experimental noise.** Here, we report the numerically computed min-entropy (solving the optimization problem in Eq.(S5)) for different regimes of noise. In particular, we show the behavior of both $H_{\min}(ABC|EF, 000)$ (upper panels) and $H_{\min}(AC|EF, 00)$ (lower panels) as a function of the bilocal inequality I_{BRGP} when we vary the indistinguishability parameter p, effectively changing Bob's measurements (see Eq.(S1)). Moreover, in each panel of the figure we consider different values of the visibility v, comparing different plots corresponding to different amounts of colored noise in the quantum states.

In Supplementary Figure 1, each plot illustrates how the min-entropy evolves as the states exhibit varying values of visibility v and a fraction of colored noise c, while systematically varying the indistinguishability parameter across its full range, $p \in [0, 1]$. The resulting min-entropy has been reported as a function of the violation of the bilocal inequality I_{BRGP}. Moreover, this analysis has been done for both cases: when the eavesdropper attempts to guess, either the outcomes of all three parties (upper panels of Supplementary Figure 1) or only the outer ones (lower panels of Supplementary Figure 1).

D. Experimental Setup and Source Characterization

In Ref. (*47*), a three-node photonic network for entanglement swapping/bilocality tests was implemented using two independent type-II SPDC sources (EPR_1, EPR_2) based on β-barium borate (BBO) crystals pumped by a 630 mW, 250 fs pulsed field at $\lambda_p = 392.5$ nm. Each source uses a 1.5 mm-thick BBO crystal for the main datasets (with 2.0 mm used in auxiliary runs to increase pair rate). The generated photon pairs are centered at 785 nm and are filtered by 3 nm (FWHM) interference filters (Semrock, near-rectangular profile); additional Semrock long-pass edge filters remove fluorescence and environmental background. To compensate spatial/temporal walk-off, each photon traverses a HWP at 45° followed by a 0.75 mm (and 1 mm for the auxiliary runs) BBO compensation crystal. Fiber-induced equatorial phase shifts between EPR_1 and EPR_2 are corrected by a tunable liquid-crystal retarder placed on photon 4. Photons 2 and 3 are coupled into single-mode fibers, temporally aligned with a motorized delay line, interfered on a 50:50 in-fiber beam splitter, and analyzed in polarization (HWPs+PBSs) to implement a probabilistic Bell-state measurement (BSM); photons 1 and 4 (Alice, Charlie) are analyzed locally with HWPs+PBSs. Measurement settings follow the optimal equatorial choices: $A_0 = C_0 = (\sigma_z + \sigma_x)/\sqrt{2}$ and $A_1 = C_1 = (\sigma_z - \sigma_x)/\sqrt{2}$, corresponding to HWP angles 11.25° and 78.75°. All outputs are coupled to single-mode fibers and detected on silicon APDs; detection events are recorded on a time-to-digital converter (id Quantique ID800). We quantify BSM quality by $p = 2P^{\mathrm{success}}_B - 1$, where

$P_B^{\rm success}$ is the probability the BSM reports the correct Bell outcome; p is estimated as $p = [CC(\infty) - CC(\Delta s)]/CC(\infty)$ via a Hong–Ou–Mandel measurement on fourfold coincidences $CC(\Delta s)$, where $CC(\Delta s)$ is the fourfold coincidence count at relative optical delay Δs, and $CC(\infty)$ denotes the coincidence counts when the delay is much larger than the photons' coherence time, i.e., when no two-photon interference occurs. In our apparatus, the maximum experimental value is $p_{\rm max} = 0.846 \pm 0.007$.

II. NUMERICAL TECHNIQUE: SCALAR EXTENSION IN THE BILOCAL NETWORK

The scalar extension method (*44*) aims to allow NPA-like relaxations in network scenarios, i.e. to approximately characterize the network quantum set, a task which is not trivial due to the non-convexity of this set. The difficulty in employing the NPA method in networks stems from one of the characterizing features of such scenarios, i.e. the presence of multiple independent sources. We can consider, for simplicity, the case of a chain network scenario, whose

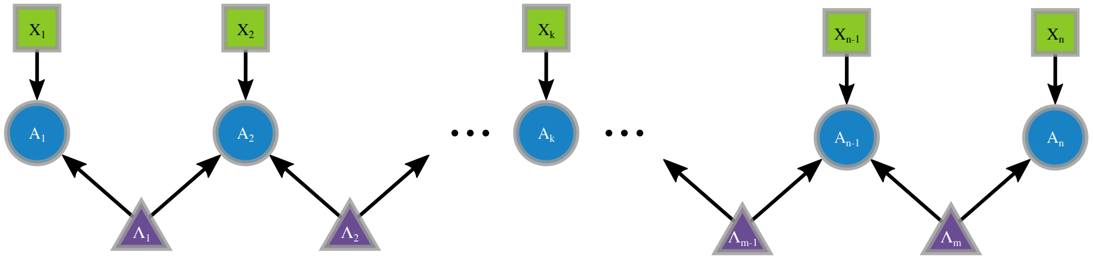

Supplementary Figure 2. **DAG of the chain network scenario.** In this scenario, m bipartite sources distribute correlations to pairs of the $n = m + 1$ parties, which perform local measurements choosing among settings described by the variables $\{X_1, \ldots, X_n\}$ and obtaining the outcomes $\{A_1, \ldots, A_n\}$.

DAG is depicted in Supplementary Figure 2. It is possible to show that behaviors arising from such structure fulfill the following condition:

$$\begin{aligned}&\sum_{a_k} p(a_1, \ldots, a_k, \ldots, a_n | x_1, \ldots, x_k, \ldots, x_n) = \\ &= p(a_1, \ldots, a_{k-1} | x_1, \ldots, x_{k-1}) p(a_{k+1}, \ldots, a_n | x_{k+1}, \ldots, x_n),\end{aligned} \tag{S6}$$

where $(a_1, \ldots, a_n)$ and $(x_1, \ldots, x_n)$ respectively denote the outcomes and the setting of the measurements performed by each party.
When we marginalize one of the non-extremal parties, the distribution factorizes, meaning that the corresponding parties are conditionally independent.
In the context of causal modeling, the independence relations can be identified by resorting to the notion of d-separation. In this case, we can say that when the path connecting two nodes contains a structure with two converging arrows (called *collider*), then the corresponding variables are conditionally independent. Therefore, observing the DAG depicted in Supplementary Figure 2, it is possible to recognize that the collider with the middle node in A_k lies in the path between the groups of outcomes $(A_1, \ldots, A_{k-1})$ and $(A_{k+1}, \ldots, A_n)$. Then, we deduce that the factorization reported in Eq.S6 encodes the conditional independence $((A_1, \ldots, A_{k-1})(A_{k+1}, \ldots, A_n)|A_k))$.
For this reason, the inability to apply the NPA method in network scenarios can be attributed to the causal separations that emerge in the presence of independent latent variables. Expressions akin to the factorized distribution in Eq.S6 are both nonlinear and non-convex, features which make it impossible to cast the characterization of the network quantum set into an SDP problem. The aim of scalar extension is indeed to overcome this obstacle by encoding the independence relations arising from the network structure in constraints which are linear in the entries of the moment matrix, then compatible with the SDP relaxations exploited by the NPA method.

A. Construction of the Method

The *scalar extension* main idea is, indeed, an extension of the moment matrix employed in the standard NPA hierarchy. In particular, we complement the set of operators $\mathcal{O}$ that generate the matrix $\mathbf{\Gamma}$ with additional operators composed of the product of operators multiplied by the expected value of other products of the operator. Then, we will extend the set $\mathcal{O}$ with operators of the form $S_i\langle S_j\rangle$ or $S_i\langle S_j\rangle\langle S_k\rangle$. Once we add such operators, there will be

factorized quantities among the new entries in the moment matrix $\tilde{\mathbf{\Gamma}}$ which allows us to set up linear relations. It is fundamental to choose the extension variable to make the factorized entries encode all independencies featured in a given scenario. At this stage, the NPA method can be applied in the usual manner by constructing a matrix $\tilde{\mathbf{\Gamma}}$, where certain entries are fixed by the observed distribution, while those corresponding to unobservable measurements are treated as variables. These variables are then optimized through a semidefinite programming (SDP) problem to verify the existence of a matrix satisfying $\tilde{\mathbf{\Gamma}} \succeq 0$. If the solution is positive, it confirms that the observed distribution belongs to the network quantum set.
Moreover, by construction, the existence of $\tilde{\mathbf{\Gamma}} \succeq 0$ implies the existence of $\mathbf{\Gamma} \succeq 0$, since the latter is a principal submatrix of the former. Instead, if we cannot find any scalar extension certificate, it means that the proposed causal explanation that we proposed is not compatible with the observed distribution. If, in addition, any matrix $\mathbf{\Gamma}$ can be found, we can also conclude that the distribution is incompatible with any measurements on a global quantum state.
To better understand the notions about the scalar extension method that we have introduced so far, we can consider the bilocal scenario as an exemplary instance of the network. Employing the d-separation criterion, we deduce that Alice's and Charlie's nodes are d-separated by Bob's node. As a consequence, all the entries of $\tilde{\mathbf{\Gamma}}$, which only contains Alice's and Charlie's operators must factorize. For instance, we can consider the moment matrix generated by the set of operators $\mathcal{O} = \{\mathbb{1}, A_{0|0}A_{0|1}, C_{0|0}C_{0|1}, \langle A_{0|0}A_{0|1}\rangle\mathbb{1}\}$.

$$\tilde{\mathbf{\Gamma}} = \begin{array}{c} \\ \mathbb{1} \\ (A_0A_1)^\dagger \\ (C_0C_1)^\dagger \\ \langle A_0A_1\rangle^*\mathbb{1} \end{array} \begin{array}{c} \begin{array}{cccc} \mathbb{1} & A_0A_1 & C_0C_1 & \langle A_0A_1\rangle\mathbb{1} \end{array} \\ \begin{pmatrix} 1 & v_1 & v_2 & v_3 \\ & 1 & v_4 & v_5 \\ & & 1 & v_6 \\ & & & v_7 \end{pmatrix} \end{array} \tag{S7}$$

where we reported only the upper triangular matrix, since $\tilde{\mathbf{\Gamma}}$ is Hermitian. A priori, we should optimize over all the variable v_i to seek the values that make the moment matrix positive semidefinite. However, the presence of the extra operator $\langle A_0A_1\rangle\mathbb{1}$ produces a series of relationships among the variables, making some dependent on others, for instance, we can deduce $v_1 = v_3$, $v_5 = v_7$, and $v_4 = v_6^*$. In particular, the latter of these equalities is crucial from a causal perspective, since it imposes the factorization $\langle A_0A_1C_0C_1\rangle = \langle A_0A_1\rangle\langle C_0C_1\rangle$ i.e. the independence constraint which arises from the network structure of the bilocal scenario. The same causal independence could be imposed by constraints like $v_4 = v_1^*v_2$ and $v_5 = |v_1|^2$. However, due to their nonlinearity, these relationships cannot be directly incorporated into an SDP problem. Consequently, we will not enforce them.

III. EXPERIMENTAL DATA PRE-PROCESSING

The experimental data obtained in (*47*) consists of the coincidence counts $N(abc|xz)$ measured in the single-photon detectors for possible measurement outcomes and settings. Given the statistical noise affecting the experimental counts, it is possible that the reconstructed probability distribution $p_{\exp}(abc|xz) = N(abc|xz)/\sum_{a,b,c} N(abc|xz)$ may violate the so-called no-signaling (NS) constraints, imposing the impossibility of signaling among space-like separated parties. As the quantum set of correlations is a subset of the NS one, when this occurrence takes place, an SDP optimization aimed at maximizing the guessing probability would not be feasible if constrained to such a distribution. To overcome this problem, we perform a "projection" of the experimentally reconstructed distributions over the NS set of correlations through the following maximum likelihood problem:

$$\begin{aligned} \max \quad & \log\mathcal{L} \equiv \log\left(\prod_{a,b,c,x,z} p(abc|xz)^{N(abc|xz)}\right) \quad \text{s.t.} \\ & \sum_{a,b,c} p(abc|xyz) = 1, \\ & \sum_a p(abc|xyz) = \sum_a p(abc|x'yz) \quad \forall x, x' \\ & \sum_c p(abc|xyz) = \sum_c p(abc|xyz') \quad \forall z, z', \\ & p(ac|xz) = p(a|x)p(c|z), \end{aligned} \tag{S8}$$

where the first constraint normalizes the variable $p(abc|xz)$, the second and the third ones impose the NS constraints, while the fourth establishes the conditional independence among the outer nodes outcomes a and c.
The solution to this problem provides the closest distribution to $p_{\exp}(abc|xz)$ that satisfies the NS constraints, which, therefore, can be safely employed to evaluate the amount of certifiable randomness from a given experimental distribution. Moreover, this pre-processing does hinder the security of our approach, as the eavesdropper cannot exploit the projection onto the no-signaling set to correlate A and C while remaining undetected. This is due to causal structures under consideration which, for all three the DE, WE, and SE scenarios, maintain the d-separation between A and C even in the presence of the eavesdropper(s).

IV. LOWER BOUNDS TO THE GUESSING PROBABILITY

In this section, we derive some lower bounds to the P_{guess}. This approach is complementary to the numerical results based on the NPA hierarchy and the scalar extension. While the latter provides a lower bound to the certifiable bits, depending on the chosen hierarchical order, here we define some minimal methods to identify a *possible* physical strategy for Eve in a generic network. This strategy can differ from the optimal one, thus providing an upper bound to the certifiable bits in a given network scenario.

A. Eavesdropping strategies in general networks

Consider a generic network defined by a DAG as in Fig. 1 in the main text. Each DAG is composed of three different kinds of nodes: the *latent* nodes, representing the network sources, the *eavesdropper* nodes, and the *observables* nodes, with (optionally) the associated *setting* node. To each latent variable, we associate a quantum state described by the density operator $\rho_\Lambda \in \mathcal{L}(\mathcal{H}_\Lambda)$. We call $\rho = \bigotimes_\Lambda \rho_\Lambda \in \mathcal{L}(\mathcal{H})$ the overall quantum state produced by the sources, where $\mathcal{H}$ is the corresponding global Hilbert space. To each observed (or eavesdropper) variable A we associate a POVM measurement described by the operators $\{\mathcal{A}^a_x\}_a$, optionally dependent on a setting x. Measurement operators relative to different nodes should be pairwise commuting. The distribution of the outcomes of the observable and eavesdropper nodes is then given by:

$$P(a_1,\dots,a_n,e_1,\dots,e_m|x_1,\dots,x_n;\rho) = \operatorname{tr}\left(\mathcal{A}^{a_1}_{x_1}\cdots\mathcal{A}^{a_n}_{x_n}\mathcal{E}^{e_1}\cdots\mathcal{E}^{e_m}\ \rho\right) \tag{S9}$$

where the directed edges determine on which part of the space $\mathcal{H}$ of the latent nodes, the measurements $\mathcal{A}^a_x$ and $\mathcal{E}^e$ act non-trivially.

To simplify the notation we will write $\mathcal{A}^{\vec{a}}_{\vec{x}} = \prod_i \mathcal{A}^{a_i}_{x_i}$ for the measurement operator on all observable nodes with settings $\vec{x}$ and outcomes $\vec{a}$.

The goal of the eavesdropper is to guess the outcome of the observable nodes for some specific values of the settings (e.g. $\vec{x}=0$ without loss of generality) with the most efficient strategy available. This corresponds to maximizing the *guessing probability* given by:

$$P_{\text{guess}}(\mathcal{A}_0) \equiv \sum_{\vec{a},\vec{b}} P(\vec{e}=\vec{a},\vec{a},\vec{b}|\vec{x}=0;\rho). \tag{S10}$$

where the variables $\vec{b}$ represent potential bits that influence the distribution, but which Eve is not interested in guessing. These would be, for example, Bob's outcomes in the case labeled AC of the main text, which corresponds to a bilocality scenario where Eve only aims to guess the bits produced by Alice and Charlie. Conversely, the certification measurements are meant to detect the action of an eavesdropper: to avoid being noticed, the eavesdropper must guarantee that:

$$\sum_{\vec{e}} P(\vec{e},\vec{a},\vec{b}|\vec{x},\vec{y};\rho) = P_Q(\vec{a},\vec{b}|\vec{x},\vec{y}), \quad \forall \vec{a},\vec{b},\vec{x},\vec{y} \tag{S11}$$

where $\vec{x},\vec{y}$ are the settings associated to the parties A and B respectively and $P_Q(\vec{a},\vec{b}|\vec{x},\vec{y}) = \operatorname{tr}\Big(\rho\cdot\mathcal{A}^{\vec{a}}_{\vec{x}}\mathcal{B}^{\vec{b}}_{\vec{y}}\Big)$ is the probability distribution of the measurements without the intervention of the eavesdropper.

In the following, we describe some generic strategies in which Eve exploits some protocol vulnerabilities.

- **Uniform guess.** The most trivial strategy available to Eve is uniformly guessing an outcome. This provides the lowest bound to the guessing probability, i.e. the utmost bound to the certifiable randomness. After an outcome

$\vec{a}$ is extracted, the conditioned probability of correctly guessing $\vec{a}$ using this basic strategy is

$$P(e=\vec{a}|\vec{a},\vec{b},0;\rho)=\frac{1}{N_{\mathcal{A}}}, \tag{S12}$$

where $N_{\mathcal{A}}$ is the number of possible outcomes of the extraction measurement $\mathcal{A}_0$. This gives the trivial bound $P_{\text{guess}}(\mathcal{A}_0)\geq P_{\text{uniform}}(\mathcal{A}_0)=N_{\mathcal{A}}^{-1}$ for the guessing probability.

In the dichotomic case, one has that $N_{\mathcal{A}}=2^N$, leading to $H_{\min}\leq N$, where N is the number of observable nodes.

- **Informed guess.** Eve can choose to guess the outcomes of $\mathcal{A}_0$ based on the knowledge of the expected quantum distribution of the extraction outcomes. Specifically, Eve should bet on the most probable result $\vec{a^*}$ for each value of $\vec{b}$.

  This leads to

$$P(e=\vec{a}|\vec{b},0;\rho)=\max_{\vec{a}} P_Q(\vec{a}|\vec{b},0;\rho)=P_Q(\vec{a^*}|\vec{b},0;\rho). \tag{S13}$$

  We call this method "informed guess", which leads to the overall guessing probability

$$P_{\text{guess}}(\mathcal{A}_0)\geq P_{\text{info}}(\mathcal{A}_0;\rho)=\sum_{\vec{b}} P(\vec{e}=\vec{a}|\vec{b},0;\rho)P_Q(\vec{b}|0;\rho)=\sum_{\vec{b}} P_Q(\vec{a^*}|\vec{b},0;\rho)P_Q(\vec{b}|0;\rho). \tag{S14}$$

  If these outcomes are uncorrelated (i.e. we have a uniform distribution $P_Q(\vec{a}|\vec{b},0;\rho)=1/N_{\mathcal{A}}$), then the guessing probability becomes the lowest possible $P_{\text{info}}(\mathcal{A}_0;\rho)=1/N_{\mathcal{A}}$. On the contrary, the more correlated the outcomes are, the more Eve can guess correctly using this basic strategy. When Eve attempts to guess all the bits produced in the nodes, then no variable $\vec{b}$ is present, and the informed guess probability reduces to $P_{\text{info}}(\mathcal{A}_0;\rho)=\max_{\vec{a}} P_Q(\vec{a}|0;\rho)$.

- **Node vulnerability.** A more sophisticated strategy consists of identifying some vulnerabilities in the protocol, which allows to perform a projective measurement $\mathcal{E}^{\vec{e}}=\mathbb{P}_{\vec{e}}$ (with distinct outcomes $\vec{e}$) that cannot be detected by the nodes.

  Then, Eve can either exploit the information gained by the knowledge of ξ or the correlations introduced by projecting the state, to define the guess probability

$$P_{\text{guess}}(\mathcal{A}_0)\geq P_{\text{info}}(\mathcal{A}_0)=\sum_{\xi}\text{tr}(\rho\mathbb{P}_{\vec{e}})P_{\text{info}}(\mathcal{A}_0;\rho_{\vec{e}}),\quad \rho_{\vec{e}}=\frac{\mathbb{P}_{\vec{e}}\rho\mathbb{P}_{\vec{e}}}{\text{tr}(\rho\mathbb{P}_{\vec{e}})}. \tag{S15}$$

  A specific example of such a mechanism can be identified when all the measurements of a node j commute, i.e. $[\mathcal{A}_x^{a_j},\mathcal{A}_{x'}^{a_j}]=0\quad\forall x,x'$. In this case, then Eve can define $\mathcal{E}^{e_j}$ as the projection of the initial state onto the shared eigenbasis of the operators $\{\mathcal{A}_x^{a_j}\}_x$. In case of degeneracies, the proper unitaries that define the shared eigenbasis must be found. Using this approach, Eve can distinguish all the outcomes, allowing her to guess with certainty the outcome of that node. This is trivially possible when a node only performs a single measurement, as in the bilocal case, where the central node Bob always performs a Bell-state projection.

V. BILOCALITY SCENARIO

Here, we discuss how to apply the strategies defined above to the case of a bilocality scenario. In the standard strategy used in this scenario, the state is generated by two sources and reads

$$\rho=\left|\Psi^-_{AB_1}\right\rangle\!\left\langle\Psi^-_{AB_1}\right|\otimes\left|\Psi^-_{B_2C}\right\rangle\!\left\langle\Psi^-_{B_2C}\right|, \tag{S16}$$

where we focus on the case of a pure state (absence of noise). The external nodes (Alice and Charlie) perform the measurements

$$\mathcal{A}_0=\mathcal{C}_0=\frac{\sigma_z+\sigma_x}{\sqrt{2}},\quad \mathcal{A}_1=\mathcal{C}_1=\frac{\sigma_z-\sigma_x}{\sqrt{2}}. \tag{S17}$$

We consider the two possible scenarios, where the central node either performs only a Bell-state measurement with four outcomes

$$\mathcal{B}^{(14)}=b_1\left|\Psi^+\right\rangle\left\langle\Psi^+\right|+b_2\left|\Psi^-\right\rangle\left\langle\Psi^-\right|+b_3\left|\Phi^+\right\rangle\left\langle\Phi^+\right|+b_4\left|\Phi^-\right\rangle\left\langle\Phi^-\right|, \tag{S18}$$

or can choose between two possible, two-outcome measurements

$$\mathcal{B}_y^{(22)}=(1-y)\sigma_z\otimes\sigma_z+y\sigma_x\otimes\sigma_x,\quad y=0,1. \tag{S19}$$

A. Minimal strategy for Eve

Eve can apply some minimal strategies without performing any measurements. The "uniform guess" bounds the maximum number of certifiable bits to be

$$H^{(14)}_{\min}(\mathcal{A}_0,\mathcal{B}^{(14)},\mathcal{C}_0) \leq 4, \qquad H^{(22)}_{\min}(\mathcal{A}_0,\mathcal{B}^{(22)}_0,\mathcal{C}_0) \leq 3,$$
$$H^{(14)}_{\min}(\mathcal{A}_0,\mathcal{C}_0) \leq 2, \qquad H^{(22)}_{\min}(\mathcal{A}_0,\mathcal{C}_0) \leq 2. \tag{S20}$$

A tighter bound can be obtained by the "informed guess" strategy without any additional operation (i.e. (S14)). Considering the bilocality measurements, one obtains an improved estimation

$$H^{(14)}_{\min}(\mathcal{A}_0,\mathcal{B}^{(14)},\mathcal{C}_0) \lesssim 3, \qquad H^{(22)}_{\min}(\mathcal{A}_0,\mathcal{B}^{(22)}_0,\mathcal{C}_0) \lesssim 2.41,$$
$$H^{(14)}_{\min}(\mathcal{A}_0,\mathcal{C}_0) \lesssim 1.41, \qquad H^{(22)}_{\min}(\mathcal{A}_0,\mathcal{C}_0) \lesssim 1.41. \tag{S21}$$

Hereafter, we discuss how an improved bound can be obtained by studying the vulnerabilities of the bilocality protocol, before using an "informed guess" strategy, as described in Eq. (S15). This is specifically relevant when considering the causal structure described in Fig.1-c of the main text.

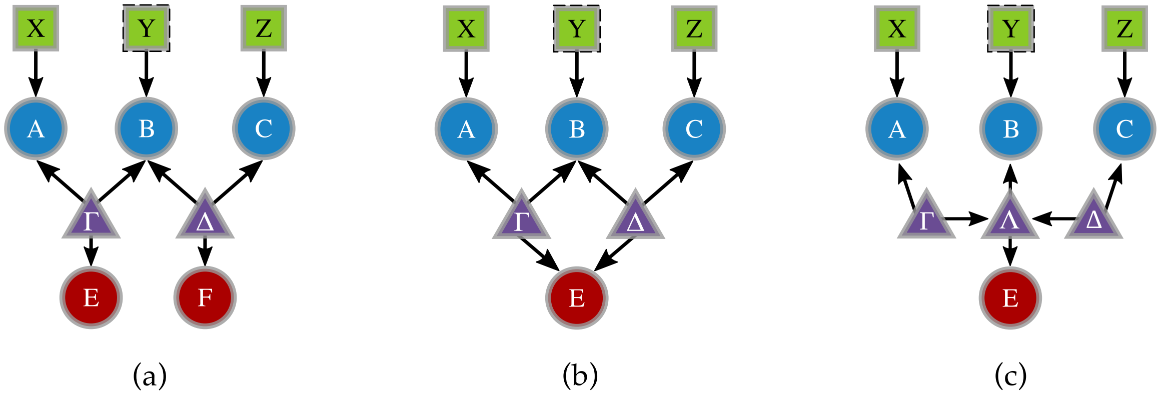

Supplementary Figure 3. **Representation of different Eavesdropping scenarios.** We reproduce here for convenience the DAGs representing the three possible eavesdropping models in the Bilocal scenario presented in the main text: the double eavesdropper (DE), the weak eavesdropper (WE), and the strong-eavesdropper (SE) scenario.

B. Strong eavesdropper scenario

In this section, we will focus on the *strong-eavesdropper* (SE) scenario. Eve can access both sources at the same time through a central latent node as shown in Fig. 3c.

We will study different strategies, based on the protocol performed by the nodes.

1. Bob's perform a Bell-state measurement (case 14)

First, we provide a minimal model that explains the strategy adopted by Eve when Bob performs a Bell-state measurement.

The vulnerability of the protocol is evident in this scenario since Bob only performs one measurement. This leaves Eve free to project onto the eigenbasis of Bob's measurement, i.e. the Bell basis, maximizing its guessing probability of Bob's node up to unity. Crucially, this operation is also compatible with the specific causal structure under analysis. The resulting state reads

$$\tilde{\rho} = \frac{1}{4}\Big(\ |\Phi^+_{AC}\rangle\langle\Phi^+_{AC}| \otimes |\Phi^+_B\rangle\langle\Phi^+_B| \ + \ |\Phi^-_{AC}\rangle\langle\Phi^-_{AC}| \otimes |\Phi^-_B\rangle\langle\Phi^-_B| \\ + \ |\Psi^+_{AC}\rangle\langle\Psi^+_{AC}| \otimes |\Psi^+_B\rangle\langle\Psi^+_B| \ + \ |\Psi^-_{AC}\rangle\langle\Psi^-_{AC}| \otimes |\Psi^-_B\rangle\langle\Psi^-_B| \ \Big), \tag{S22}$$

where we used the decomposition

$$\left|\Psi^-_{AB_1}\right\rangle\left|\Psi^-_{B_2C}\right\rangle=\frac{1}{2}\left(-\left|\Phi^+_{AC}\right\rangle\left|\Phi^+_{B}\right\rangle+\left|\Phi^-_{AC}\right\rangle\left|\Phi^-_{B}\right\rangle+\left|\Psi^+_{AC}\right\rangle\left|\Psi^+_{B}\right\rangle-\left|\Psi^-_{AC}\right\rangle\left|\Psi^-_{B}\right\rangle\right). \tag{S23}$$

This new state clearly satisfies $P_Q(a,b_0,b_1,c|\rho,x,z)=P_Q(a,b_0,b_1,c|\tilde{\rho},x,z)$. Now, Eve can guess Bob's outcome with unit probability. What about the outcomes of Alice and Charlie? For those nodes, we can use the "informed guess" scheme conditioned on Eve's outcome. We have

$$\begin{aligned}
P_Q(a\vec{b}c|\Phi_+,x=0,z=0) &= \frac{1}{4}[1+(-1)^{a+c}]\delta^0_{b_0}\delta^0_{b_1},\\
P_Q(a\vec{b}c|\Phi_-,0,0) &= \frac{1}{4}\delta^0_{b_0}\delta^1_{b_1},\\
P_Q(a\vec{b}c|\Psi_+,0,0) &= \frac{1}{4}\delta^1_{b_0}\delta^0_{b_1},\\
P_Q(a\vec{b}c|\Psi_-,0,0) &= \frac{1}{4}[1-(-1)^{a+c}]\delta^1_{b_0}\delta^1_{b_1}.
\end{aligned} \tag{S24}$$

Using Eq.(S15), we get that $\max_{a\vec{b}c}P_Q(a\vec{b}c|\psi,0,0)$ is 1/2 when $\psi=\Phi_+,\Psi_-$ and 1/4 otherwise. Averaging over the four Bell states, we obtain

$$\begin{aligned}
P^{(14)}_{\text{guess}}(\mathcal{A}_0,\mathcal{B}^{(14)},\mathcal{C}_0)\geq P_{\text{info}}(\mathcal{A}_0,\mathcal{B}^{(14)},\mathcal{C}_0)=0.375,\\
P^{(14)}_{\text{guess}}(\mathcal{A}_0,\mathcal{C}_0)\geq P_{\text{info}}(\mathcal{A}_0,\mathcal{C}_0)=0.375.
\end{aligned} \tag{S25}$$

In terms of certifiable bits, one has:

$$H^{(14)}_{\min}(\mathcal{A}_0,\mathcal{B}^{(14)},\mathcal{C}_0)\lesssim 1.41,\qquad H^{(14)}_{\min}(\mathcal{A}_0,\mathcal{C}_0)\lesssim 1.41. \tag{S26}$$

Eve's specific strategy consists of predicting Bob's outcome with certainty, then performing an informed guess on Alice's and Charlie's bits. This explains why $H^{(14)}_{\min}(\mathcal{A}_0,\mathcal{B}^{(14)},\mathcal{C}_0)=H^{(14)}_{\min}(\mathcal{A}_0,\mathcal{C}_0)\lesssim 1.41$. Similarly, it also explains why Eve's guessing probability for the outmost nodes (Alice and Charlie) is not improved compared to the basic strategy of Eq.(S21).

2. *Bob performs separable measurements (case 22)*

In this case, we can use the fact that the two operators performed by Bob commute

$$[\mathcal{B}^{(22)}_0,\mathcal{B}^{(22)}_1]=[\sigma_z\otimes\sigma_z,\sigma_x\otimes\sigma_x]=0, \tag{S27}$$

to apply a strategy similar to the case of the Bell-state measurement. Specifically, due to the commutation relations the two operators share an eigenbasis, which is given by the Bell basis itself. Eve can thus apply the same strategy previously discussed, by performing a Bell-state measurement and feeding Alice, Bob, and Charlie with the state $\tilde{\rho}_4$ of Eq. (S22) rather than the initial state ρ_4. In this way, Eve will automatically know the outcome of Bob's measurement and, at the same time, will not change the results of Alice, Bob, and Charlie's measurements. Analogously to the previous case, using Eq. (S15), we get:

$$H^{(22)}_{\min}(\mathcal{A}_0,\mathcal{B}^{(22)}_0,\mathcal{C}_0)\lesssim 1.41,\qquad H^{(22)}_{\min}(\mathcal{A}_0,\mathcal{C}_0)\lesssim 1.41. \tag{S28}$$

Instead we can consider a scenario in which Bob's measurements do not commute, for instance, when he measures $B_0=\sigma_x\otimes\sigma_x$ and $B_1=\frac{\sigma_x+\sigma_z}{\sqrt{2}}\otimes\frac{\sigma_x+\sigma_z}{\sqrt{2}}$. In this case, Eve no longer has a single basis to optimally guess Bob's outcome in both settings, therefore, choosing $y=0$ or $y=1$ yields to different amounts of certified randomness, as reported in Fig.4. Moreover, we notice that the min-entropy corresponding to $y=0$ surpasses the one certified in the (2,2) choice, indicating how measurements commutation negatively affect the amount of certified randomness within a given scenario.

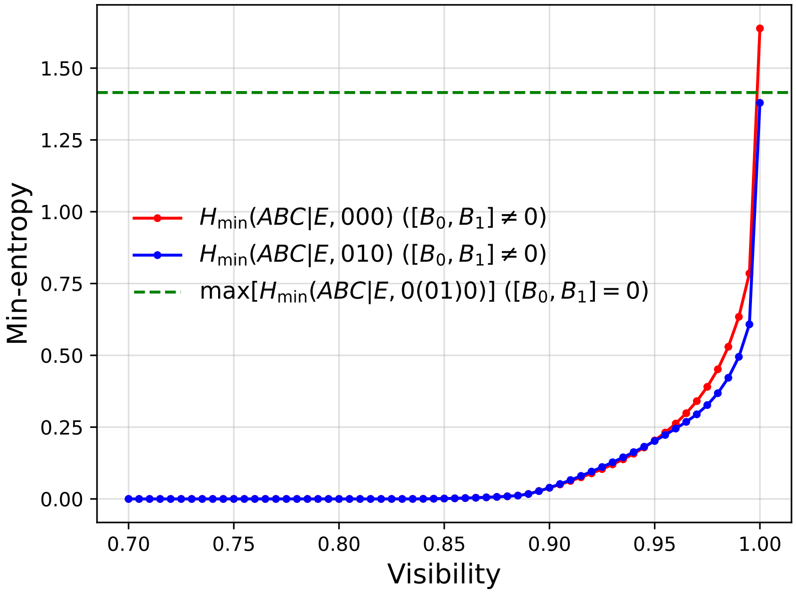

Supplementary Figure 4. **Certified randomness for non-commuting Bob's measurements.** In this figure, we report the amount of certified randomness in a scenario in which Bob's measurements do not commute. In particular, we consider a strong-eavesdropper scenario in which the parties perform the measurements described in the main text as the (2,2) choice, except for Bob which measures $B_0 = \sigma_x \otimes \sigma_x$ and $B_1 = \frac{\sigma_x+\sigma_z}{\sqrt{2}} \otimes \frac{\sigma_x+\sigma_z}{\sqrt{2}}$. The min-entropies corresponding to Bob's settings $y = 0$ and $y = 1$ are respectively depicted as blue and red dots. For comparison, the maximum amount of randomness certified in the case of $[B_0, B_1] = 0$ (i.e. right panel of Fig.3a of the main text) is reported as a green dashed line.

C. Double Eavesdropper scenario

In this section, we comment on the causal structure shown in Fig. 3a, i.e. the "double-eavesdropper" scenario. This corresponds to assuming an additional constraint on Eve, who now cannot act jointly on the qubits produced by the two sources.

The main strategy described in the previous sections consists of Eve projecting Bob's qubits on the Bell basis, which strongly relies on the ability to access both sources at the same time. When the eavesdropper can only access the sources separately, that strategy becomes unavailable, explaining why a higher number of random bits can be certified. Specifically, the numerical optimization confirms that in the case of maximal visibility $\nu = 1$, Eve's best strategy consists of simply using the "informed guess" protocol of Eq.(S14), thus leading to the certified bits reported in Eq.(S21), which coincide with the results of the numerical simulations, confirming that this is indeed the optimal strategy.

D. Weak Eavesdropper randomness using self-testing

Self-testing protocols for networks, and specifically for the bilocality scenario, have been demonstrated in ref.(*77*) Using this idea, we can certify that a specific quantum strategy was employed in the network, based only on the observable probability distribution. This in turn will allow us to exclude the possibility of an active eavesdropper strategy accessing the sources, leaving the *informed guess* strategy as the optimal one. We will first consider the tilted strategy described in Eq.13 of the main text.

Consider the bilocality scenario with dichotomic measurements $A^a_x \in \mathcal{L}(\mathcal{H}_A)$ and $C^c_z \in \mathcal{L}(\mathcal{H}_C)$, and a four-outcome measurement $B^b_{B_1 B_2} \in \mathcal{L}(\mathcal{H}_{B_1} \otimes \mathcal{H}_{B_2})$, and let us call $\langle A_x C_y \rangle_b = \sum_{ac} (-1)^{a+c} p(a, c|b)$ the correlator on A and C conditioned on having outcome b for B. Similarly, we can define the postselected state $\rho^b_{AC} \in \mathcal{L}(\mathcal{H}_A \otimes \mathcal{H}_C)$, as the effective state shared by A and C for each outcome b of the central node. We will start by stating the following lemma.

Lemma 1. *Assume that for a given b we have*

$$\langle A_0 C_0 \rangle_b = 0 \qquad \langle A_1 C_0 \rangle_b = \langle A_0 C_1 \rangle_b = \cos\delta \qquad \langle A_1 C_1 \rangle_b = -\sin 2\delta \tag{S29}$$

and $\langle A_0\rangle_b = \langle A_1\rangle_b = \langle C_0\rangle_b = \langle C_1\rangle_b = 0$. *Then, up to local unitary* $U_A \otimes V_C$*, the postselected state is* $\rho^b_{AC} = \left|\Phi^+_{A'C'}\right\rangle\left\langle\Phi^+_{A'C'}\right| \otimes \rho_{\text{junk}}$ *and the corresponding measurements on* A *and* C *are of the form* $A_x \otimes \mathbb{1}_{\text{junk}}$ *and* $C_z \otimes \mathbb{1}_{\text{junk}}$ *respectively, where*

$$\begin{aligned} A_0 &= \sigma_z \quad A_1 = \sigma_x \cos\delta - \sigma_z \sin\delta \\ C_0 &= \sigma_x \quad C_1 = \sigma_z \cos\delta - \sigma_x \sin\delta, \end{aligned} \tag{S30}$$

and the subscript "junk" denotes additional unused degrees of freedom.

Proof. A proof can be derived directly from the self-testing result contained in section D of the Supplemental Material contained in ref.(*51*), noticing that Eq.(S29) maximizes the inequality I_δ. □

Applying slight variations of Lemma 1 repeatedly, we can obtain self-testing results for each Bell state $\{\left|\Phi^b\right\rangle\}_b = \{\left|\Phi^+\right\rangle, \left|\Psi^+\right\rangle, \left|\Phi^-\right\rangle, \left|\Psi^-\right\rangle\}$, based on having postselected correlations of the form:

$$\begin{aligned} &\langle A_0 C_1\rangle_{b=(b_0,b_1)} = (-1)^{b_0}\cos\delta && \langle A_1 C_0\rangle_{b=(b_0,b_1)} = (-1)^{b_1}\cos\delta \\ &\langle A_0 C_0\rangle_b = 0 && \langle A_1 C_1\rangle_{b=(b_0,b_1)} = \delta_{b_0,b_1}(-1)^{b_0}\sin(2\delta) \end{aligned} \tag{S31}$$

By using this we can conclude the following:

Lemma 2. *Given a bilocality scenario with a four-outcome measurement* B^b*, measurements* A_x *and* C_z *satisfying Eq.*(S31)*, there exist two completely positive and unital maps* $\mathcal{C}_1 : \mathcal{L}(\mathcal{H}_{B_1}) \to \mathcal{L}(\mathcal{H}_{B_1'}), \mathcal{C}_2 : \mathcal{L}(\mathcal{H}_{B_2}) \to \mathcal{L}(\mathcal{H}_{B_2'})$*, such that* $(\mathcal{C}_1 \otimes \mathcal{C}_2)(B^b_{B_1B_2}) = \left|\Phi^b_{B_1'B_2'}\right\rangle\left\langle\Phi^b_{B_1'B_2'}\right|$.

Moreover, there exist local maps $\mathcal{L}_1 : \mathcal{L}(\mathcal{H}_{AB_1}) \to \mathcal{L}(\mathcal{H}_{A'B_1})$ *and* $\mathcal{L}_2 : \mathcal{L}(\mathcal{H}_{B_2C}) \to \mathcal{L}(\mathcal{H}_{B_2'C'})$ *such that* $\mathcal{L}_1(\rho_{AB_1}) = \left|\Phi^+_{A'B_1'}\right\rangle\left\langle\Phi^+_{A'B_1'}\right| \otimes \rho_{\text{junk}}$ *and* $\mathcal{L}_2(\sigma_{B_2C}) = \left|\Phi^+_{B_2'C'}\right\rangle\left\langle\Phi^+_{B_2'C'}\right| \otimes \rho_{\text{junk}}$ *where* ρ_{AB_1} *and* σ_{B_2C} *are the initial states of the two sources.*

Proof. To prove it, we can use the fact that having a distribution like the one in Eq.(S31) self-tests for a strategy with measurements that are unitarily equivalent to Eq.(S30) and states such that $U_A \otimes V_C \rho^b_{AC} U_A^\dagger \otimes V_C^\dagger = \left|\Phi^b_{AC}\right\rangle\left\langle\Phi^b_{AC}\right| \otimes \rho_{\text{junk}}$ for some local unitary $U_A \otimes V_C$. Following ref. (*77*), we can then define the maps $\mathcal{C}_1, \mathcal{C}_2$ as the ones generated by the states $\alpha_{B_1'B_1} = \text{Tr}_{\text{junk}}(U_A \rho_{AB_1} U_A^\dagger)$ and $\gamma_{B_2B_2'} = \text{Tr}_{\text{junk}}(U_C \rho_{B_2C} U_C^\dagger)$. Indeed, when applied to $B^b_{B_1B_2}$ we have

$$(\mathcal{C}_1 \otimes \mathcal{C}_2)(B^b_{B_1B_2}) = \text{Tr}_{B_1B_2}(\alpha_{B_1'B_1}\gamma_{B_2B_2'}\ B^b_{B_1B_2}) = \\ \text{Tr}_{\text{junk}}\left(U_{AC}\,\text{Tr}_{B_1B_2}(\rho_{AB_1}\rho_{B_2C}\ B^b_{B_1B_2})U^\dagger_{AC}\right) = \left|\Phi^b_{B_1'B_2'}\right\rangle\left\langle\Phi^b_{B_1'B_2'}\right| \tag{S32}$$

where, to simplify the notation we are using only the subscripts to keep track of the spaces the operators act on. Also, it is direct to see that $\mathcal{C}_1, \mathcal{C}_2$ are CP since $\alpha_{B_1'B_1}$ and $\gamma_{B_2B_2'}$ are positive operators, and it is unital since $\mathcal{C}_1(\mathbb{1}_{B_1}) = \text{Tr}_{B_1}(\alpha_{B_1'B_1}) = \sum_b \text{Tr}_{B_1B_2B_2'}(\alpha_{B_1'B_1}\gamma_{B_2B_2'}B^b_{B_1B_2}) = \sum_b \text{Tr}_{B_2'}\left(\left|\Phi^b_{B_1'B_2'}\right\rangle\left\langle\Phi^b_{B_1'B_2'}\right|\right) = \mathbb{1}_{B_1'}$, and similarly for $\mathcal{C}_2$.

To prove that the source states are equivalent to Bell states call $\mathcal{B}_1$ and $\mathcal{B}_2$ the maps associated with the CJ operators $\Lambda_1 = \text{Tr}_{B_2}(V_{B_1'}B^0_{B_1B_2}\sigma_{B_2B_1'}V^\dagger_{B_1'})$ and $\Lambda_2 = \text{Tr}_{B_1}(U_{B_2'}B^0_{B_1B_2}\rho_{B_2'B_1}U^\dagger_{B_2'})$ respectively, and we define the maps $\mathcal{L}_1, \mathcal{L}_2$ as $\mathcal{L}_1 = \mathcal{U} \otimes \mathcal{B}_1$ and $\mathcal{L}_2 = \mathcal{B}_2 \otimes \mathcal{V}$, where $\mathcal{U}$ and $\mathcal{V}$ are the unitary transformations given by U_A and V_C respectively. In this way, we have that:

$$\mathcal{L}_1(\rho_{AB_1}) = \text{Tr}_{B_1}\left(U_A\rho_{AB_1}U_A^\dagger\Lambda_{1\,B_1B_1'}\right) = U_AV_{B_1'}\,\text{Tr}_{B_1B_2}\left(\rho_{AB_1}B^0_{B_1B_2}\sigma_{B_2B_1'}\right)U_A^\dagger V^\dagger_{B_1'} = \\ U_AV_{B_1'}\rho^0_{AB_1'}U_A^\dagger V^\dagger_{B_1'} = \left|\Phi^0_{A'B_1'}\right\rangle\left\langle\Phi^0_{A'B_1'}\right| \otimes \rho_{\text{junk}} \tag{S33}$$

and similarly for $\mathcal{L}_2(\sigma_{B_2C})$. □

Using the lemma above, we can conclude that the optimal strategy corresponds to the one we called *informed guess.*

Proposition 1. *For the scenarios represented by the DAGs in Fig. 3a-3b, if the observed distribution satisfies Eq.*(S31)*, the optimal strategy for the eavesdropper gives* $H_{\min}(ABC|Ex = 0, z = 0) = -\log(\max p(abc|x = 0, z = 0))$*, and* $H_{\min}(AC|Ex = 0, z = 0) = -\log(\sum_b p(b)\max p(ac|b, x = 0, z = 0))$.

Proof. Using lemma 2 we have that $(\mathcal{L}_1 \otimes \mathcal{L}_2)(\rho_{ABC}) = \left|\Phi^+_{AB_1}\right\rangle\left\langle\Phi^+_{AB_1}\right| \otimes \left|\Phi^+_{B_2C}\right\rangle\left\langle\Phi^+_{B_2C}\right| \otimes \rho_{\text{junk}}$, where $= \rho_{ABC} = \text{Tr}_E(\rho_{ABCE})$. This means that we have $\rho_{ABCE} = \rho_{ABC} \otimes \rho_E$, and the eavesdropper cannot acquire any information by measuring her subsystem. □

This result indeed coincides with the maximum found with the numerical optimization in the case of the DE scenario (see Fig.6 in the main text), but it is also valid for the WE scenario, for which the optimization technique we employed cannot be used. Specifically, this allows us to reach 4 bits of certified randomness in both cases.

VI. SINGLE-PARTY RANDOMNESS CERTIFICATION

An interesting figure of merit to consider within our framework is the amount of randomness that can be certified from a single party, as already studied in multipartite scenarios (*26*, *78*).
In our framework, this task is achieved by changing the objective function of the SDP optimization to the corresponding single-party guessing probability:

$$G(N, E, x) = \sum_n p(n, e = (n)|x), \tag{S34}$$

corresponding to a situation in which the eavesdropper tries to guess the measurement outcomes n of an arbitrary node of the network $N \in \{A, B, C\}$. In particular, in the bilocal scenario, it is interesting to consider the amount of certifiable randomness from the central node and one of the external nodes (for instance, A, since, by symmetry, the results for C are identical).

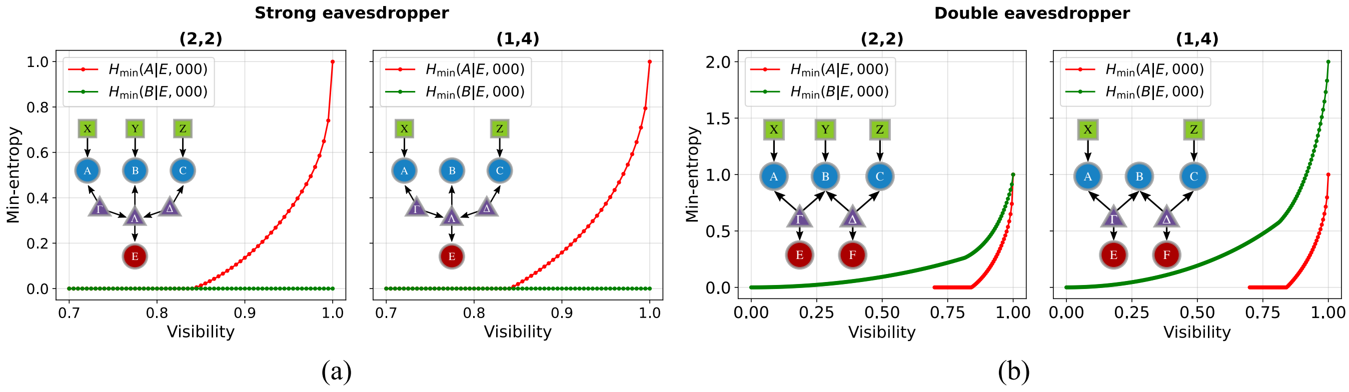

Supplementary Figure 5. **Single-party certified randomness for different configurations of the bilocal scenario.** In this figure, we report the min-entropy corresponding to the single-party randomness certification in the bilocal scenario in both the strong- (panel **(a)**) and double- (panel (b)) eavesdropping scenarios, and considering both the (2,2) and (1,4) central node measurement strategies. For each of the four combinations of the eavesdropping scenarios and measurement strategies, we report the amount of certified randomness from the outcomes of either the central node (green dots) or one of the outer nodes (red dots), in this case chosen to be Alice. This quantities are reported as a function of the visibility of the states distributed by the two independent sources, which have the form $\rho_\Gamma = \rho_\Delta = v\left|\Psi^-\right\rangle\!\left\langle\Psi^-\right| + (1-v)\frac{\mathbb{1}}{4}$, where v is the visibility parameter.

In Fig. 5, we report the results for both the measurement choices considered in the work, i.e., (2,2) and (1,4), and both double- and strong-eavesdropper attacking strategies. Within the SE scenario, as expected, the outcomes of the central node's measurements are always correctly guessed by the eavesdropper, thereby preventing to certify any randomness independently from the chosen measurement strategy. Different results are obtained, instead, when considering the DE strategy. Firstly, as expected, Bob's outcomes cease to be completely predictable as in the SE scenario, showing a non-zero amount of randomness in the entire range of visibility. This feature is compatible with the observation made for Fig. (3) of the main text, since, also in this case, a portion of such randomness is due to the fact that, within the DE scenario, neither of the two eavesdroppers has access to both the independent sources. Since the central node performs measurements on subsystems of the states distributed by both the sources, the eavesdroppers will always lack a part of the information that defines Bob's outcome, for any visibility. Therefore, in the DE scenario, it is possible to certify up to 1 and 2 bits of randomness in the (2,2) and (1,4), respectively. Concerning the randomness certified from an outer node, the DE and SE scenarios produce equivalent results. In fact, having access to one or both

the sources does not make any difference, as all the information needed to guess the outcomes of an external node comes from the adjacent source. Also, independently of the measurement strategy adopted by the central node, it is possible to certify up to 1 bit of randomness from Alice's outcomes. Finally, it is worth to underline that, except for the complete predictability of Bob's outcomes in the SE scenario, all the other results reach their corresponding maximum amount of certifiable randomness. Moreover, in all these cases, the certifiable randomness at maximum visibility is compatible with the explicit eavesdropping strategy presented in Section IV of the Supplementary information, which, when maximal randomness is certified, corresponds to a complete ignorance about the outcomes, leaving as the only possible strategy for the eavesdropper to uniformly guess them.

REFERENCES

1. A. Einstein, B. Podolsky, N. Rosen, Can quantum-mechanical description of physical reality be considered complete? *Phys. Rev.* **47**, 777–780 (1935).

2. J. S. Bell, On the einstein podolsky rosen paradox. *Phys. Phys. Fizika* **1**, 195–200 (1964).

3. N. Brunner, D. Cavalcanti, S. Pironio, V. Scarani, S. Wehner, Bell nonlocality. *Rev. Mod. Phys.* **86**, 419–478 (2014).

4. N. Gisin, Physics. Quantum nonlocality: How does nature do it? *Science* **326**, 1357–1358 (2009).

5. S. Beigi, R. König, Simplified instantaneous non-local quantum computation with applications to position-based cryptography. *New J. Phys.* **13**, 093036 (2011).

6. H. Buhrman, R. Cleve, S. Massar, R. De Wolf, Nonlocality and communication complexity. *Rev. Mod. Phys.* **82**, 665–698 (2010).

7. C. Portmann, R. Renner, Security in quantum cryptography. *Rev. Mod. Phys.* **94**, 025008 (2022).

8. J. Yin, Y.-H. Li, S.-K. Liao, M. Yang, Y. Cao, L. Zhang, J.-G. Ren, W.-Q. Cai, W.-Y. Liu, S.-L. Li, R. Shu, Y.-M. Huang, L. Deng, L. Li, Q. Zhang, N.-L. Liu, Y.-A. Chen, C.-Y. Lu, X.-B. Wang, F. Xu, J.-Y. Wang, C.-Z. Peng, A. K. Ekert, J.-W. Pan, Entanglement-based secure quantum cryptography over 1,120 kilometres. *Nature* **582**, 501–505 (2020).

9. T. Jennewein, C. Simon, G. Weihs, H. Weinfurter, A. Zeilinger, Quantum cryptography with entangled photons. *Phys. Rev. Lett.* **84**, 4729–4732 (2000).

10. S. Pirandola, U. L. Andersen, L. Banchi, M. Berta, D. Bunandar, R. Colbeck, D. Englund, T. Gehring, C. Lupo, C. Ottaviani, J. L. Pereira, M. Razavi, J. S. Shaari, M. Tomamichel, V. C. Usenko, G. Vallone, P. Villoresi, P. Wallden, Advances in quantum cryptography. *Adv. Opt. Photonics* **12**, 1012–1236 (2020).

11. S. Pironio, A. Acín, S. Massar, A. B. de la Giroday, D. N. Matsukevich, P. Maunz, S. Olmschenk, D. Hayes, L. Luo, T. A. Manning, C. Monroe, Random numbers certified by bell's theorem. *Nature* **464**, 1021–1024 (2010).

12. O. Nieto-Silleras, S. Pironio, J. Silman, Using complete measurement statistics for optimal device-independent randomness evaluation. *New J. Phys.* **16**, 013035 (2014).

13. A. Acín, L. Masanes, Certified randomness in quantum physics. *Nature* **540**, 213–219 (2016).

14. A. Acín, S. Massar, S. Pironio, Randomness versus nonlocality and entanglement. *Phys. Rev. Lett.* **108**, 100402 (2012).

15. A. Acín, S. Pironio, T. Vértesi, P. Wittek, Optimal randomness certification from one entangled bit. *Phys. Rev. A* **93**, 040102 (2016).

16. E. Woodhead, J. Kaniewski, B. Bourdoncle, A. Salavrakos, J. Bowles, A. Acín, R. Augusiak, Maximal randomness from partially entangled states. *Phys. Rev. Res.* **2**, 042028 (2020).

17. Y. Liu, Q. Zhao, M.-H. Li, J.-Y. Guan, Y. Zhang, B. Bai, W. Zhang, W.-Z. Liu, C. Wu, X. Yuan, H. Li, W. J. Munro, Z. Wang, L. You, J. Zhang, X. Ma, J. Fan, Q. Zhang, J.-W. Pan, Device-independent quantum random-number generation. *Nature* **562**, 548–551 (2018).

18. S. Gómez, A. Mattar, E. S. Gómez, D. Cavalcanti, O. J. Farías, A. Acín, G. Lima, Experimental nonlocality-based randomness generation with nonprojective measurements. *Phys. Rev. A* **97**, 040102 (2018).

19. M.-H. Li, X. Zhang, W.-Z. Liu, S.-R. Zhao, B. Bai, Y. Liu, Q. Zhao, Y. Peng, J. Zhang, Y. Zhang, W. J. Munro, X. Ma, Q. Zhang, J. Fan, J.-W. Pan, Experimental realization of device-independent quantum randomness expansion. *Phys. Rev. Lett.* **126**, 050503 (2021).

20. L. K. Shalm, Y. Zhang, J. C. Bienfang, C. Schlager, M. J. Stevens, M. D. Mazurek, C. Abellán, W. Amaya, M. W. Mitchell, M. A. Alhejji, H. Fu, J. Ornstein, R. P. Mirin, S. W. Nam, E. Knill, Device-independent randomness expansion with entangled photons. *Nat. Phys.* **17**, 452–456 (2021).

21. A. J.-M. Seguinard, A. Piveteau, P. Mironowicz, M. Bourennane, Experimental certification of more than one bit of quantum randomness in the two inputs and two outputs scenario. *New J. Phys.* **25**, 113022 (2023).

22. I. Agresti, D. Poderini, L. Guerini, M. Mancusi, G. Carvacho, L. Aolita, D. Cavalcanti, R. Chaves, F. Sciarrino, Experimental device-independent certified randomness generation with an instrumental causal structure. *Commun. Phys.* **3**, 110 (2020).

23. R. Colbeck, Quantum and relativistic protocols for secure multi-party computation. arXiv:0911.3814 [quant-ph] (2009).

24. F. J. Curchod, M. Johansson, R. Augusiak, M. J. Hoban, P. Wittek, A. Acín, Unbounded randomness certification using sequences of measurements. *Phys. Rev. A* **95**, 020102 (2017).

25. J.-D. Bancal, L. Sheridan, V. Scarani, More randomness from the same data. *New J. Phys.* **16**, 033011 (2014).

26. E. Woodhead, B. Bourdoncle, A. Acín, Randomness versus nonlocality in the Mermin-Bell experiment with three parties. *Quantum* **2**, 82 (2018).

27. F. Grasselli, G. Murta, H. Kampermann, D. Bruß, Boosting device-independent cryptography with tripartite nonlocality. *Quantum* **7**, 980 (2023).

28. E. Polino, L. Villegas-Aguilar, D. Poderini, N. Walk, F. Ghafari, M. T. Quintino, A. Lyasota, S. Rogge, R. Chaves, G. J. Pryde, E. G. Cavalcanti, N. Tischler, S. Slussarenko, Experimental quantum randomness enhanced by a quantum network. arXiv:2412.16973 [quant-ph] (2024).

29. A. Tavakoli, A. Pozas-Kerstjens, M.-X. Luo, M.-O. Renou, Bell nonlocality in networks. *Rep. Prog. Phys.* **85**, 056001 (2022).

30. D. Poderini, I. Agresti, G. Marchese, E. Polino, T. Giordani, A. Suprano, M. Valeri, G. Milani, N. Spagnolo, G. Carvacho, R. Chaves, F. Sciarrino, Experimental violation of n-locality in a star quantum network. *Nat. Commun.* **11**, 2467 (2020).

31. R. Chaves, G. Moreno, E. Polino, D. Poderini, I. Agresti, A. Suprano, M. R. Barros, G. Carvacho, E. Wolfe, A. Canabarro, R. W. Spekkens, F. Sciarrino, Causal networks and freedom of choice in bell's theorem. *PRX Quantum* **2**, 040323 (2021).

32. A. Suprano, D. Poderini, E. Polino, I. Agresti, G. Carvacho, A. Canabarro, E. Wolfe, R. Chaves, F. Sciarrino, Experimental genuine tripartite nonlocality in a quantum triangle network. *PRX Quantum* **3**, 030342 (2022).

33. E. Polino, D. Poderini, G. Rodari, I. Agresti, A. Suprano, G. Carvacho, E. Wolfe, A. Canabarro, G. Moreno, G. Milani, R. W. Spekkens, R. Chaves, F. Sciarrino, Experimental nonclassicality in a causal network without assuming freedom of choice. *Nat. Commun.* **14**, 909 (2023).

34. F. Andreoli, G. Carvacho, L. Santodonato, M. Bentivegna, R. Chaves, F. Sciarrino, Experimental bilocality violation without shared reference frames. *Phys. Rev. A* **95**, 062315 (2017).

35. N. D'Alessandro, B. Polacchi, G. Moreno, E. Polino, R. Chaves, I. Agresti, F. Sciarrino, Machine-learning-based device-independent certification of quantum networks. *Phys. Rev. Res.* **5**, 023016 (2023).

36. N.-N. Wang, A. Pozas-Kerstjens, C. Zhang, B.-H. Liu, Y.-F. Huang, C.-F. Li, G.-C. Guo, N. Gisin, A. Tavakoli, Certification of non-classicality in all links of a photonic star network without assuming quantum mechanics. *Nat. Commun.* **14**, 2153 (2023).

37. X.-M. Gu, L. Huang, A. Pozas-Kerstjens, Y.-F. Jiang, D. Wu, B. Bai, Q.-C. Sun, M.-C. Chen, J. Zhang, S. Yu, Q. Zhang, C.-Y. Lu, J.-W. Pan, Experimental full network nonlocality with independent sources and strict locality constraints. *Phys. Rev. Lett.* **130**, 190201 (2023).

38. N.-N. Wang, C. Zhang, H. Cao, K. Xu, B.-H. Liu, Y.-F. Huang, C.-F. Li, G.-C. Guo, N. Gisin, T. Kriváchy, M.-O. Renou, Experimental genuine quantum nonlocality in the triangle network. arXiv:2401.15428 [quant-ph] (2024).

39. D. J. Saunders, A. J. Bennet, C. Branciard, G. J. Pryde, Experimental demonstration of nonbilocal quantum correlations. *Sci. Adv.* **3**, e1602743 (2017).

40. G. Carvacho, E. Roccia, M. Valeri, F. B. Basset, D. Poderini, C. Pardo, E. Polino, L. Carosini, M. B. Rota, J. Neuwirth, S. F. Covre da Silva, A. Rastelli, N. Spagnolo, R. Chaves, R. Trotta, F. Sciarrino, Quantum violation of local causality in an urban network using hybrid photonic technologies. *Optica* **9**, 572–578 (2022).

41. C. M. Lee, M. J. Hoban, Towards device-independent information processing on general quantum networks. *Phys. Rev. Lett.* **120**, 020504 (2018).

42. E. Wolfe, A. Pozas-Kerstjens, M. Grinberg, D. Rosset, A. Acín, M. Navascués, Quantum inflation: A general approach to quantum causal compatibility. *Phys. Rev. X* **11**, 021043 (2021).

43. P. Sekatski, S. Boreiri, N. Brunner, Partial self-testing and randomness certification in the triangle network. *Phys. Rev. Lett.* **131**, 100201 (2023).

44. A. Pozas-Kerstjens, R. Rabelo, Ł. Rudnicki, R. Chaves, D. Cavalcanti, M. Navascués, A. Acín, Bounding the sets of classical and quantum correlations in networks. *Phys. Rev. Lett.* **123**, 140503 (2019).

45. M. Z. Żukowski, A. Zeilinger, M. A. Horne, A. K. Ekert, "Event-ready-detectors" Bell experiment via entanglement swapping. *Phys. Rev. Lett.* **71**, 4287–4290 (1993).

46. C. Branciard, D. Rosset, N. Gisin, S. Pironio, Bilocal versus nonbilocal correlations in entanglement-swapping experiments. *Phys. Rev. A* **85**, 032119 (2012).

47. G. Carvacho, F. Andreoli, L. Santodonato, M. Bentivegna, R. Chaves, F. Sciarrino, Experimental violation of local causality in a quantum network. *Nat. Commun.* **8**, 14775 (2017).

48. M. Navascués, S. Pironio, A. Acín, A convergent hierarchy of semidefinite programs characterizing the set of quantum correlations. *New J. Phys.* **10**, 073013 (2008).

49. S. Pironio, S. Massar, Security of practical private randomness generation. *Phys. Rev. A* **87**, 012336 (2013).

50. C. Dhara, G. Prettico, A. Acín, Maximal quantum randomness in bell tests. *Phys. Rev. A* **88**, 052116 (2013).

51. L. Wooltorton, P. Brown, R. Colbeck, Tight analytic bound on the trade-off between device-independent randomness and nonlocality. *Phys. Rev. Lett.* **129**, 150403 (2022).

52. R. Bhavsar, S. Ragy, R. Colbeck, Improved device-independent randomness expansion rates using two sided randomness. *New J. Phys.* **25**, 093035 (2023).

53. M. Zukowski, A. Zeilinger, H. Weinfurter, Entangling photons radiated by independent pulsed sources. *Ann. N. Y. Acad. Sci.* **755**, 91–102 (1995).

54. K. Azuma, S. E. Economou, D. Elkouss, P. Hilaire, L. Jiang, H.-K. Lo, I. Tzitrin, Quantum repeaters: From quantum networks to the quantum internet. *Rev. Mod. Phys.* **95**, 045006 (2023).

55. Z.-D. Li, R. Zhang, X.-F. Yin, L.-Z. Liu, Y. Hu, Y.-Q. Fang, Y.-Y. Fei, X. Jiang, J. Zhang, L. Li, N.-L. Liu, F. Xu, Y.-A. Chen, J.-W. Pan, Experimental quantum repeater without quantum memory. *Nat. Photonics* **13**, 644–648 (2019).

56. Y.-A. Chen, Q. Zhang, T.-Y. Chen, W.-Q. Cai, S.-K. Liao, J. Zhang, K. Chen, J. Yin, J.-G. Ren, Z. Chen, S.-L. Han, Q. Yu, K. Liang, F. Zhou, X. Yuan, M.-S. Zhao, T.-Y. Wang, X. Jiang, L. Zhang, W.-Y. Liu, Y. Li, Q. Shen, Y. Cao, C.-Y. Lu, R. Shu, J.-Y. Wang, L. Li, N.-L. Liu, F. Xu, X.-B. Wang, C.-Z. Peng, J.-W. Pan, An integrated space-to-ground quantum communication network over 4,600 kilometres. *Nature* **589**, 214–219 (2021).

57. S.-K. Liao, W.-Q. Cai, J. Handsteiner, B. Liu, J. Yin, L. Zhang, D. Rauch, M. Fink, J.-G. Ren, W.-Y. Liu, Y. Li, Q. Shen, Y. Cao, F.-Z. Li, J.-F. Wang, Y.-M. Huang, L. Deng, T. Xi, L. Ma, T. Hu, L. Li, N.-L. Liu, F. Koidl, P. Wang, Y.-A. Chen, X.-B. Wang, M. Steindorfer, G. Kirchner, C.-Y. Lu, R. Shu, R. Ursin, T. Scheidl, C.-Z. Peng, J.-Y. Wang, A. Zeilinger, J.-W. Pan, Satellite-relayed intercontinental quantum network. *Phys. Rev. Lett.* **120**, 030501 (2018).

58. I. Šupić, J.-D. Bancal, Y. Cai, N. Brunner, Genuine network quantum nonlocality and self-testing. *Phys. Rev. A* **105**, 022206 (2022).

59. R. Chaves, Polynomial bell inequalities. *Phys. Rev. Lett.* **116**, 010402 (2016).

60. D. Centeno, E. Wolfe, Distinguishing quantum causal scenarios with indistinguishable classical analogs: The significance of intermediate latents. *Phys. Rev. A* **112**, 042206 (2025).

61. B. Hensen, H. Bernien, A. E. Dréau, A. Reiserer, N. Kalb, M. S. Blok, J. Ruitenberg, R. F. L. Vermeulen, R. N. Schouten, C. Abellán, W. Amaya, V. Pruneri, M. W. Mitchell, M. Markham, D. J. Twitchen, D. Elkouss, S. Wehner, T. H. Taminiau, R. Hanson, Loophole-free bell inequality violation using electron spins separated by 1.3 kilometres. *Nature* **526**, 682–686 (2015).

62. W. Rosenfeld, D. Burchardt, R. Garthoff, K. Redeker, N. Ortegel, M. Rau, H. Weinfurter, Event-ready bell test using entangled atoms simultaneously closing detection and locality loopholes. *Phys. Rev. Lett.* **119**, 010402 (2017).

63. M. Navascués, S. Pironio, A. Acín, Bounding the set of quantum correlations. *Phys. Rev. Lett.* **98**, 010401 (2007).

64. R. F. Werner, Quantum states with einstein-podolsky-rosen correlations admitting a hidden-variable model. *Phys. Rev. A* **40**, 4277–4281 (1989).

65. Q.-C. Sun, Y.-F. Jiang, B. Bai, W. Zhang, H. Li, X. Jiang, J. Zhang, L. You, X. Chen, Z. Wang, Q. Zhang, J. Fan, J.-W. Pan, Experimental demonstration of non-bilocality with truly independent sources and strict locality constraints. *Nat. Photonics* **13**, 687–691 (2019).

66. F. Andreoli, G. Carvacho, L. Santodonato, R. Chaves, F. Sciarrino, Maximal qubit violation of n-locality inequalities in a star-shaped quantum network. *New J. Phys.* **19**, 113020 (2017).

67. P. Lauand, D. Poderini, R. Rabelo, R. Chaves, Quantum non-classicality in the simplest causal network. arXiv:2404.12790 [quant-ph] (2024).

68. J. Jiang, J. Zhang, X. Sun, Quantum-to-quantum bernoulli factory problem. *Phys. Rev. A* **97**, 032303 (2018).

69. Y. Liu, J. Jiang, P. Zhu, D. Wang, J. Ding, X. Qiang, A. Huang, P. Xu, J. Zhang, G. Tian, X. Fu, M. Deng, C. Wu, X. Sun, X. Yang, J. Wu, General quantum bernoulli factory: Framework analysis and experiments. *Quantum Sci. Technol.* **6**, 045025 (2021).

70. F. Hoch, T. Giordani, L. Castello, G. Carvacho, N. Spagnolo, F. Ceccarelli, C. Pentangelo, S. Piacentini, A. Crespi, R. Osellame, E. F. Galvão, F. Sciarrino, Modular quantum-to-quantum bernoulli factory in an integrated photonic processor. *Nat. Photonics* **19**, 12–19 (2025).

71. G. Rodari, F. Hoch, A. Suprano, T. Giordani, E. Negro, G. Carvacho, N. Spagnolo, E. F. Galvão, F. Sciarrino, Polarization-encoded photonic quantum-to-quantum bernoulli factory based on a quantum dot source. *Sci. Adv.* **10**, eado6244 (2024).

72. A. Broadbent, J. Fitzsimons, E. Kashefi, "Universal blind quantum computation," in *2009 50th annual IEEE symposium on foundations of computer science* (IEEE, 2009), pp. 517–526. https://doi.org/10.1109/FOCS.2009.36.

73. B. Polacchi, D. Leichtle, L. Limongi, G. Carvacho, G. Milani, N. Spagnolo, M. Kaplan, F. Sciarrino, E. Kashefi, Multi-client distributed blind quantum computation with the qline architecture. *Nat. Commun.* **14**, 7743 (2023).

74. B. Polacchi, D. Leichtle, G. Carvacho, G. Milani, N. Spagnolo, M. Kaplan, E. Kashefi, F. Sciarrino, Experimental verifiable multiclient blind quantum computing on a qline architecture. *Phys. Rev. Lett.* **134**, 200603 (2025).

75. K. Mattle, H. Weinfurter, P. G. Kwiat, A. Zeilinger, Dense coding in experimental quantum communication. *Phys. Rev. Lett.* **76**, 4656–4659 (1996).

76. A. Cabello, A. Feito, A. Lamas-Linares, Bell's inequalities with realistic noise for polarization-entangled photons. *Phys. Rev. A* **72**, 052112 (2005).

77. M. O. Renou, J. Kaniewski, N. Brunner, Self-testing entangled measurements in quantum networks. *Phys. Rev. Lett.* **121**, 250507 (2018).

78. Y. Li, Y. Xiang, X.-D. Yu, H. C. Nguyen, O. Gühne, Q. He, Randomness certification from multipartite quantum steering for arbitrary dimensional systems. *Phys. Rev. Lett.* **132**, 080201 (2024).
